# A misleading tail: A long-term study of reptile responses to multiple disturbances undermined by a change in surveying techniques

**DOI:** 10.1371/journal.pone.0305518

**Published:** 2024-06-14

**Authors:** Maldwyn John Evans, Christopher MacGregor, David Lindenmayer

**Affiliations:** Fenner School of Environment and Society, The Australian National University, Canberra, ACT, Australia; Universidad Miguel Hernandez de Elche, SPAIN

## Abstract

Long-term ecological monitoring is crucial to understanding the complex dynamics of ecosystems, communities, and populations. Despite this, monitoring data are lacking or rare for the vast majority of biodiversity. Here we report the results of 19 years (2003–2022) of continuous annual monitoring of reptile species at Booderee National Park (BNP) on the east coast of south-eastern Australia. We tested the effects of time, habitat type, fire, and climate on detections of five reptile species. Our study revealed declines in detections of two skink species over time (*Lampropholis delicata* and *Ctenotus taeniolatus*), which we suspect was partly driven by weather conditions influencing activity of these species. We also identified broad vegetation type associations for two congeneric species with *L*. *delicata* being associated with forested sites, and *Lampropholis guichenoti* associated with more shrubby sites. Our results also demonstrated a clear association between *Cryptophis nigrescens* and *L*. *delicata* and fire, with the probabilities of detection of both species decreasing with time since fire in the short term. At about the midway point of our study (in 2011), we were forced to make a change in the way our data were collected. The change heavily influenced our findings, and so breached the integrity of the time series in our dataset. We acknowledge that a simple but crucial step to mitigate this breach would have been to conduct calibration that allowed subsequent analysis to control for a change in field survey methodology. Whilst improvements in the effectiveness of field survey methods might be possible through new technologies, it is crucial to maintain the integrity of long-term datasets as data collection continues.

## Introduction

Long-term ecological monitoring is crucial if we are to understand the complex dynamics of ecosystems, communities, and populations [1, 2]. Long-term data allow the study of ecological processes that occur over long time periods and the quantification of biotic responses to ecosystem change [1, 3]. They are particularly important during the current human-driven environmental crisis, when disturbances driven by climate change, landscape modification, resource exploitation, and invasive species are impacting biodiversity in novel and unprecedented ways [4–6]. Long-term data can provide information to mitigate the effects of disturbances on biodiversity [2].

Despite the importance of long-term data, they are lacking or rare for the vast majority of ecosystems and biodiversity. This is unsurprising given the challenges in collecting ecological data over long periods of time. Most ecological research is conducted over short timescales dictated by the limited length of funding cycles [7]. Ongoing collection of data, therefore, usually requires dedicated work by those researchers involved to secure long-term funding [8]. Furthermore, funding for consistent collection of data over long periods is often not as attractive to funding bodies as short-term research is often perceived as being more novel and innovative [8].

Reptiles are a group in need of high-quality long-term data. This is because 20% of reptile species are threatened with extinction globally; more species than birds or mammals [9]. Threats to reptiles include agricultural expansion, logging, urbanisation, invasive species, and hunting [9]. Given that reptiles are ectothermic, climate change is also considered a significant threat [10]. Impacts of climate change on reptiles include skewing of populations of species with temperature-dependent sex ratios [11, 12], altering the locations and availability of optimal nesting habitat [13], and physiological stress due to perturbations in temperature [14].

Despite these threats, long-term data are rare for the majority of reptile species [but see; 15–21]. Gathering data on a range of species in a reptile community is challenging. Data collection can be highly influenced by the field methods employed. For example, Michael et al. [22] showed that artificial refuges (corrugated steel, roof tiles, and timber) were more likely to detect certain species than active search techniques and vice versa. Further, most reptile community studies are unable to reliably collect information on rare or cryptic species [23], leaving long-term data on only relatively common and easily detected species.

Here we report the results of 19 years (2003–2022) of continuous annual monitoring of reptile species at 108 sites at Booderee National Park (BNP) on the east coast of south-eastern Australia. BNP is an important reserve for some of Australia’s rare species [24] and the monitoring program is an example of a strong and enduring partnership between scientific researchers and park managers [25]. BNP supports more than 725 native plant species which occur in a broad range of vegetation types from sedgeland and heathlands, to woodlands and rainforest [24]. Research and monitoring at BNP has included work on birds [26], mammals [27], invasive plants [28], and reintroduced native mammals [29]. The reptile fauna at the park is rich and varied with 15 species detected in the 19 years of the study.

BNP has experienced a range of ecological disturbances in the last 20 years, which likely impact reptile species in the park. Quantifying how reptiles have responded to these disturbances is fundamental to their ongoing conservation. For example, there have been extensive control efforts for invasive plants such as bitou bush (*Chrysanthemoides monilifera ssp*. *rotundata*), which is a species that can alter vegetation structure [30]. There has also been intensive feral animal control, with a particular emphasis on reducing the numbers of the red fox (*Vulpes vulpes*) in BNP [31]. The removal of this predator has precipitated a trophic cascade with an increase in native herbivores [27], which have, in turn, impacted vegetation structure [31, 32]. Fire is a key ecological process in BNP, with all major vegetation types impacted by fire [33]. Finally, Australia has an extremely variable climate [34], characterized by periods of drought interspersed with heavy rain [35]. BNP, therefore, has experienced a range of climatic conditions during the 19-year duration of this study.

In this investigation, we quantified the trajectories of five native reptile species (Table 1); delicate skink (*Lampropholis delicata*), common garden skink (*Lampropholis guichenoti*), copper-tailed skink (*Ctenotus taeniolatus*), jacky dragon (*Amphibolurus muricatus*), and small-eyed snake (*Cryptophis nigrescens*). These species were common enough to provide sufficient data for analysis, whilst also differing in their life-history traits. For example, *L*. *delicata* is most often associated with forest habitats [36, 37], whilst the closely-related species *L*. *guichenoti* can be found in habitats with less overstorey such as backyard gardens and urban areas [38]. Likewise, *C*. *taeniolatus* is often associated with dry forest and grassy box woodland [24]. *C*. *nigrescens* is a nocturnal snake which feeds almost exclusively on skinks and *A*. *muricatus* is a terrestrial and semi-arboreal lizard which is larger than the other three species of skinks in our study [24] (Table 1).

**Table 1:** Species analysed in the two time periods and their life-history traits, diet, average length, and habitat associations. The two time period columns indicate in which species we were able to analyse in each period. Species information from Michael et al. [52], Cogger [38], Brag et al. [36], Lunney et al. [53], Taylor et al. [54], and Howard et al. [37].

Family	Species	Life-history	Diet	Length	Habitat association	Time period 1 (‘03-‘10)	Time period 2 (‘11-‘22)
Scincidae	Delicate skink *Lampropholis delicata*	Terrestrial, oviparous, diurnal.	Small invertebrates	51 mm (snout-vent)	Forest.	Π	Π
	Garden skink *Lampropholis guichenoti*	Terrestrial, oviparous, diurnal.	Small invertebrates	48 mm (snout-vent)	Open woodland.	Π	
	Copper-tailed skink *Ctenotus taeniolatus*	Terrestrial, oviparous, diurnal.	Small invertebrates	80 mm (snout-vent)	Dry forest, open woodland.	Π	
Agamidae	Jacky dragon *Amphibolurus muricatus*	Terrestrial and semi arboreal, oviparous, diurnal.	Small invertebrates	120 mm (snout-vent)	Forest, open woodland	Π	
Elapidae	Small-eyed snake *Cryptophis nigrescens*	Terrestrial and saxicolous, viviparous, nocturnal.	Skinks	500 mm	Dry forest.		Π

At approximately the midway point of our study (in 2011), we were forced to change the way in which reptile data were collected (see Methods section). After this change, there was a marked change in detections of reptiles, impacting the integrity of our long-term data.

Here, we first sought to answer a series of questions designed to test how reptile species may have responded to the environmental perturbations that have occurred during the 19 years of continuous reptile monitoring at BNP. We examine how reptile detections may have been influenced by our change of monitoring method. We use this example to showcase the implications of a method change that likely breached the integrity of our time-series dataset.

### Q1. Were there associations between reptile captures and vegetation type, did species detections change over time, and do trajectories differ between vegetation types?

BNP supports a variety of broad vegetation types, from sedgelands and heathlands to woodlands, forests, and rainforests [26, 39]. This presented an opportunity to monitor reptile population trends in different environments. We expected the forest specialist *L*. *delicata* to be more common in forest sites than others [36, 37], whereas we anticipated that *C*. *taeniolatus* would be less common in forest sites than sites in other vegetation types [36]. Given that species are likely associated with certain vegetation types, we also hypothesized that there may be differences in trajectories over time within each vegetation type. For example, vegetation structure may have been altered following fire [32] favouring some species more than others. Changes in climate, including dry periods such as the Millenium drought [35], also may have impacted food availability for reptiles over time. For example, the abundance of soil invertebrates is likely to decline in periods of drought [40].

### Q2. Did fire and differences in climate during the study period influence reptile captures?

Many reptile species are sensitive to fire [41–44]. Previous research at BNP revealed that *L*. *delicata* and *C*. *nigrescens* were associated with recently sites burnt [44] and that *C*. *taeniolatus* was more abundant with an increasing frequency of past fires [45]. We expected, therefore, that we would see similar responses for these species in this longer-term investigation.

Modelling has predicted that under climate change scenarios, reptiles are likely to decline over time [46, 47]. Declines of reptiles in Europe have been linked to climate and habitat change [48]. Australia experienced the Millennium Drought from 2001–2009, which coincided with the first years of our study [35]. However, during the latter years of the study, Australia experienced increased rainfall and milder temperatures; conditions associated with the La Niña climatic conditions in the Southern Pacific [49]. Whilst drier and hotter conditions might result in declines of some species, wetter and milder conditions may lead to lower probabilities of detection of some species [50]. On this basis, at the outset of this study, we were agnostic about reptile responses over time in our study.

### Q3. Did the change in survey methodology breach the integrity of our long-term data?

Reptile detections have been shown to be significantly influenced by surveying technique [22, 23, 51]. Therefore, given the enforced change in survey methodology in 2011, we expected that some species detections to differ before and after the change.

## Materials and methods

### Study area

We conducted our research at Booderee National Park (BNP), a 6600 ha IUCN Category II protected area located approximately 150 km south of Sydney in south-eastern Australia [24]. In June, the area receives on average of just over 150 mm of rain with an average temperature of 16.5°C (Austral Winter) and in January it receives an average of 95 mm of rain with an average temperature of 25.0°C (Austral Summer).

### Approvals

Monitoring in this study received animal ethics approval through The Australian National University (Approval numbers C.R.E.60.09, A2012/49, A2015/60, A2018/58, and A2021/52). Research at Booderee National Park was contracted by the Commonwealth Director of National Parks. All species sampled are protected under the Australian Commonwealth Environment Protection and Biodiversity Conservation Act 1999, however, no critically endangered, endangered, or vulnerable species were sampled.

### Survey design

**Fig 1:**
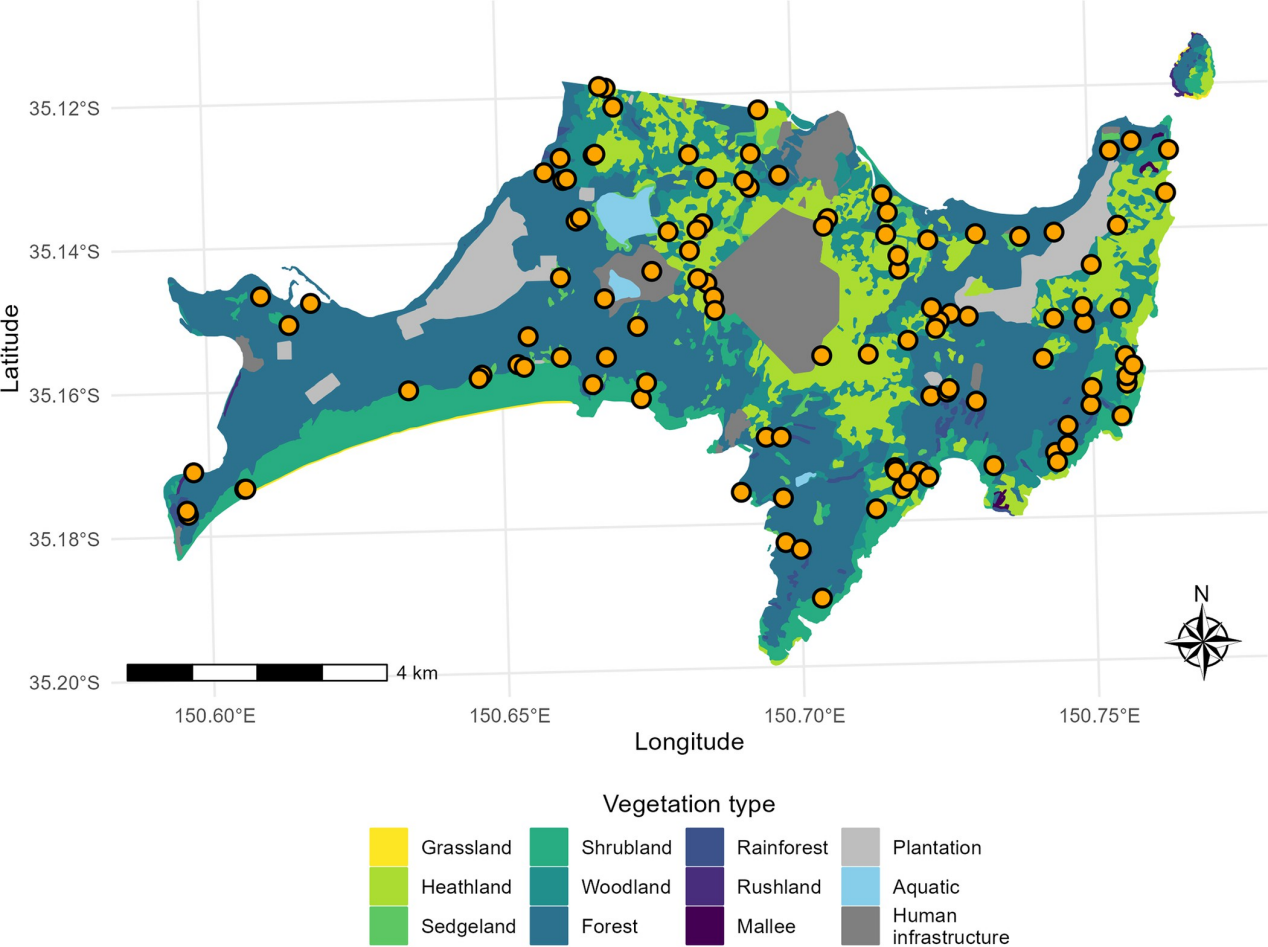
The spatial location of Booderee National Park, and its land cover types as mapped by Taws [39]. Orange points show locations of study sites. The map has been created using the ‘ggplot2’ package [56] in R version 4.2.3 using data from Taws [39].

We established 108 permanent survey sites in the seven key vegetation types at Booderee NP (Fig 1): warm temperate rainforests, forests, woodlands, heathlands, shrublands, swamps, and sedgelands [for a detailed breakdown of the survey design, including site selection, see 45, 55]. Our survey sites were also stratified by fire history at the time of establishment, based on four classes of time since the last fire (0–10 years, 11–20 years, 21–30 years, and > 30 years) [45]. Each of our sites comprised a 100m long transect, on which we conducted surveys of a variety of taxa, including reptiles [45].

### Modification of reptile monitoring

In 2011 which was the approximate midway point of our study, we were forced to change the way reptile data were collected. We made this change for several reasons:-

1. We observed that animals in pitfalls were prone to predation from birds of prey and from the brown antechinus *Antechinus stuartii* (a carnivorous marsupial).
2. A successful reduction in populations of the exotic predator *V*. *vulpes* at BNP, which, in turn, led to an increase in native herbivores [27]. At several sites, these native herbivores consumed the damp course material used in the construction of the drift fences between pairs of pitfall traps along the transect at each field site.
3. The deadly (to humans) funnelweb spider (*Atrax spp*., *Hadronyche spp*.) was frequently caught in pitfall traps in forest and woodland sites, presenting a safety risk to field researchers.

As a result of changes in methodology, our reptile monitoring data consisted of detections in two time periods using two distinct methodologies.

1. From 2003, we established pitfall traps at each site from 0m to 100m in 20 m intervals along each site transect (S1 Table in S1 File). We used a plastic drift fence connecting the 0 m and 20 m pitfalls, the 80m and 100 m pitfalls, and the 40 m, 50 m, and 60 m pitfalls and bucket. We conducted surveys at various times of the year up until 2010, with surveys post 2004 occurring every year in Summer and every second year in Spring (S2 and S3 Tables and S2 Fig in S1 File). During most surveys, we opened traps for three consecutive days and nights, with variations occurring during adverse weather [45].
2. From 2011, we replaced the pitfall traps with a series of artificial substrates at each site. The substrates comprised of four roof tiles, two 1m^2^ sheets of corrugated iron (one placed over the other), and four wooden sleepers that were placed at both the 20m and 80m points along each of our 108 permanent field sites (S2 Fig in S1 File).

#### Vegetation types

Extensive vegetation mapping conducted by Taws [39] showed there has been no demonstrable change in vegetation cover in BNP since 1976 (Fig 1 and Table 2). Lindenmayer et al. [26] also noted that there has been little change in vegetation communities since the surveys by Taws [39].

**Table 2:** Predictor variables used in analysis.

Name	Symbol	Details	Varies over	Type	Min	Max	Variable used in modelling
*Year*	*Y*_*t*_	The year of the survey (2002 to 2022).	Time	Continuous	2003	2022	Rescaled (mean = 0, SD = 1).
Season	*S*_*t*_	The season in which the survey took place.	Time	Factor	-	-	Three levels: Autumn, Summer, Spring.
*Vegetation type*	*V*_*i*_	Vegetation type mapped by Taws [39].	Site	Factor	-	-	Six factor levels: Heathland, Sedgeland, Woodland, Shrubland, Forest, Rainforest.
*Fire frequency*	*FP*_*i*,*t*_	The fire frequency in the 30 years prior to the survey.	Time and site	Continuous	0	5	Rescaled (mean = 0, SD = 1).
*Time since fire*	*FT*_*i*,*t*_	The time since the last fire at the site.	Time and site	Continuous	26 days	44,818 days (122.8 years)	Rescaled (mean = 0, SD = 1).
*Minimum temperature*	*TMIN*_*t*_	Mean minimum monthly temperature for the year.	Time	Continuous	13.43°C	18.8°C	Rescaled (mean = 0, SD = 1).
*Maximum temperature*	*TMAX*_*t*_	Mean maximum monthly temperature for the season in which the survey was taken.	Time	Continuous	19.23°C	25.67°C	Rescaled (mean = 0, SD = 1).
*Precipitation*	*P*_*t*_	Mean monthly precipitation for the season in which the survey was taken.	Time	Continuous	30.3 mm	216.77 mm	Rescaled (mean = 0, SD = 1).
*Precipitation in previous year*	*PPR*_*t*_	Mean monthly precipitation in the year prior to the survey	Time	Continuous	56.53 mm	220.47 mm	Rescaled (mean = 0, SD = 1)
*Survey methodology*	*M*_*t*_	The survey methodology.	Time	Factor	-	-	Two levels: Early (2002–2010) Late (2011–2022)

#### Fire history

Booderee National Park has a well-documented history of fire. There have been 259 fires between 1957 and 2021 including five large-scale (> 500 ha) wildfires in 1962 and 1973 (x2), 2003 and 2017 [33]. The majority of fires have been low-intensity prescribed burns < 10 ha and wildfires <10 ha. These fires are often patchy, with unburned patches within the total area of the burn [57].

We used two fire variables in our models. The first, *fire frequency*, was the number of fires that occurred on a given site in the 30 years prior [26]. The second fire variable, *time since fire*, we defined as the time since the last fire at a given site [26].

#### Climate

We downloaded climate-history data from the Australian Bureau of Meteorology (Jervis Bay [Point Perpendicular Station]) [58]. We used mean maximum and minimum monthly temperatures and mean monthly precipitation data from 2001 to 2022. From these data, we calculated the mean values of the three variables (maximum monthly temperature, minimum monthly temperatures, monthly precipitation) for each of four seasons (Austral Spring, Summer, Winter, and Autumn) for each year. This enabled us to associate climate data in the season in which surveys were taken. We also calculated mean monthly precipitation for the year preceding the survey, as reptiles are known to increase reproduction following higher than average rainfall [59].

### Statistical analysis

We constructed a series of Bayesian binomial generalized linear mixed models to test the effects of time, vegetation, fire, and climate predictors on reptile detection during our surveys. For Q1 and Q2, we treated the two sampling time periods as separate analyses, given their very different survey techniques. We used presence/absence data for reptiles with sufficient data to construct robust statistical models (> 5% of surveys). This enabled us to analyse the responses of the five species of reptiles as outlined above (Table 1).

We constructed models using the ‘brms’ package [60, 61] in R [62], assuming a Bernoulli error distribution. We conducted a model selection procedure for each of our questions using leave-one-out cross validation information criterion scores (LOOIC) [63] to determine the most parsimonious model for each question (Table 3); that is, the simplest model within two LOOIC scores of the best-fitting model [63, 64]. We included a ‘season’ variable (*S*_*t*_) in all models to account for the capture rates that might occur at differing times of year in which sampling took place. Using the model from question one as an example, we assumed that:

$$logit(\psi_{i,t})=\beta_{0}+\beta_{1}S_{t}+\beta_{2}Y_{t}+\beta_{3}Y_{t}^{2}+u_{i},$$

Where *ψ*_*i*,*t*_ is the probability of occurrence at site *i*, and year *t*,*β*_0_ is the intercept, and *β*_1_ to *β*_3_ are the associated regression coefficients representing the linear effects of the season and time and the quadratic effect of time. For the temporal variables *Year* (*Y*_*t*_), *Time since fire* (*FT*_*i*_,_*t*_), *Precipitation* (*P*_*t*_), and *Precipitation in previous year* (*PPR*_*t*_), we included both linear and quadratic terms when constructing models. For Q2, to determine the most parsimonious models between the fire and climate components, we fitted a further model with the combination of variables from both fire and climate best-fit models.

**Table 3:** Models fitted in our model selection procedure. Regression coefficients *β*_1_ to *β*_*n*_ are excluded from the model formulae. See Table 2 for variable abbreviations.

Question	Models fitted
Null model	*~ S*_*t*_ *+u*_*i*_
1. Were there associations between reptile captures and vegetation type, did species detections change over time, and do trajectories differ between vegetation types?	~ $S_{t}+Y_{t}+Y_{t}^{2}+u_{i}$, *~ S*_*t*_*+V*_*i*_ + *u*_*i*_, ~ $S_{t}+V_{i}+Y_{t}+Y_{t}^{2}+u_{i}$, ~ $S_{t}+V_{i}+Y_{t}+Y_{t}^{2}+V_{i}Y_{t}+V_{i}{Y_{t}^{2}+u}_{i}$
2. Did fire and differences in climate during the study period influence reptile captures?	Fire component: *~ S*_*t*_ *+ FP*_*i*,*t*_ + *u*_*i*_, ~ $S_{t}+{FT}_{i,t}+{FT}_{i,t}^{2}$ + *u*_*i*_, ~ $S_{t}+{{FP}_{i,t}+FT}_{i,t}+{FT}_{i,t}^{2}$ + *u*_*i*_, *~ St + FP*_*i*,*t*_ + *V*_*i*_ + *u*_*i*_, ~ $S_{t}+{FT}_{i,t}+{FT}_{i,t}^{2}$ + *V*_*i*_ + *u*_*i*_, ~ $S_{t}+{{FP}_{i,t}+FT}_{i,t}+{FT}_{i,t}^{2}$ + *V*_*i*_ + *u*_*i*_, *~ S*_*t*_ *+ FP*_*i*,*t*_ + *V*_*i*_ + *FP*_*i*_,_*t*_*V*_*i*_+ *u*_*i*_, ~ $S_{t}{+FT}_{i,t}+{FT}_{i,t}^{2}$ + $V_{i}+{FT}_{i,t}V_{i}+{FT}_{i,t}^{2}V_{i}+u_{i}$
	Climate component: ~*S*_*t*_ + *TMIN*_*t*_ + *u*_*i*_, ~*S*_*t*_ + *TMAX*_*t*_ + *u*_*i*_, *~ S*_*t*_ *+ P*_*t*_ *+ u*_*i*_, ~*S*_*t*_ + *TMIN*_*t*_ + *TMAX*_*t*_ *+u*_*i*_, ~*S*_*t*_ + *TMIN*_*t*_ + *P*_*t*_ +*u*_*i*_, ~*S*_*t*_ + *TMAX*_*t*_ + *P*_*t*_ + *u*_*i*_, ${\sim S}_{t}+{TMIN}_{t}+{TMAX}_{t}+{P_{t}+u}_{i}$, ${\sim S}_{t}+{TMIN}_{t}+{{PPR}_{t}+u}_{i}$, $\sim S_{t}+{TMAX}_{t}+{{PPR}_{t}+u}_{i}$, ~ $S_{t}+P_{t}+{{PPR}_{t}+u}_{i}$, ~ $S_{t}+{TMIN}_{t}+{TMAX}_{t}+{{PPR}_{t}+u}_{i}$, $\sim S_{t}+{TMIN}_{t}+P_{t}+{{PPR}_{t}+u}_{i}$, ${\sim S}_{t}+{TMAX}_{t}+P_{t}+{{PPR}_{t}+u}_{i}$, ${\sim S}_{t}+{TMIN}_{t}+{TMAX}_{t}+{P_{t}+{PPR}_{t}+u}_{i}$ Combined fire and climate: Model selection of the combined variables of the most parsimonious fire and climate models.
3. Did the change in survey methodology breach the integrity of our long-term data?	All of the formulae above plus the same model set with the addition of *M*_*t*_, plus a final model with the interaction of the best-fit model terms and *M*_*t*_

To answer Q3, we pooled the data from the two time periods for *L*. *delicata*, the only species with sufficient data for analysis in both survey periods. We then tested all the models in Table 3 using these pooled data, as well as the same models with the addition of the *M*_*t*_ term. This allowed us to test whether survey period variable had a strong effect on reptile detections. We also compared this best-model fit to a model with *M*_*t*_ as an interaction with the other predictors in the model. This allowed us to demonstrate whether there were contrasting responses to the survey methods in each of the predictor variables in the best-fit model.

In all models, we included site-level random effect *u*_*i*_ which allowed for dependence of repeated measures between years. We fitted all models with normal priors specifying four chains, 2,000 iterations, including 1000 warm-up/burn-in iterations. We used the Gelman-Rubin $\hat{R}$ statistic [65], and examined trace plots to assess whether the chains showed adequate mixing. We used R version 4.2.3 [62] for all analysis, including the ‘brms’ [60, 61] and ‘tidyverse’ packages [66].

## Results

We recorded 15 species of reptiles over 19 years and 3911 surveys in our study (Table 4). However, we detected the vast majority (N = 10 species) only infrequently with insufficient data to enable subsequent detailed statistical analyses for either the early or the late survey period. The five species we detected sufficiently often to facilitate statistical analyses included three skinks, a jacky dragon lizard, and a snake (Table 4).

**Table 4:** Species detected in field surveys. *Individuals* is the total sum of detected individuals of that species, *Surveys* is the number of surveys in which the species was detected, and % is the percentage of surveys in which that species was detected (See S2 and S3 Tables in S1 File for information about surveys undertaken).

			Detections 2003–2010 (1482 surveys)			Detections 2011–2022 (2429 surveys)		
Family	Species	Common name	Individuals	Surveys	%	Individuals	Surveys	%
Scincidae	*Lampropholis delicata*	Delicate skink	1508	814	54.93%	952	562	23.14%
	*Lampropholis guichenoti*	Garden skink	301	205	13.83%	29	17	0.70%
	*Ctenotus taeniolatus*	Copper-tailed skink	126	95	6.41%	24	19	0.78%
	*Acritoscincus platynotus*	Red-throated skink	86	62	4.18%	100	62	2.55%
	*Eulamprus quoyii*	Eastern water skink	50	41	2.77%	47	32	1.32%
	*Cyclodomorphus michaeli*	Eastern she-oak skink	29	28	1.89%	66	58	2.39%
	*Tiliqua scincoides*	Common blue-tongued skink	12	12	0.81%	11	11	0.45%
	*Cryptoblepharus virgatus*	Striped snake-eyed skink	3	2	0.13%	2	2	0.08%
Pygopodidae	*Pygopus lepidopodus*	Common scaly-foot	7	7	0.47%	2	2	0.08%
Agamidae	*Amphibolurus muricatus*	Jacky dragon	123	106	7.15%	6	5	0.21%
Elapidae	*Hemiaspis signata*	Marsh snake	10	10	0.67%	56	43	1.77%
	*Cryptophis nigrescens*	Small-eyed snake	6	6	0.40%	634	429	17.66%
	*Pseudechis porphyriacus*	Red-bellied black snake	1	1	0.07%	31	30	1.24%
	*Pseudonaja textilis*	Eastern brown snake	0	0	0.00%	4	4	0.16%
	*Morelia spilota*	Carpet python	0	0	0.00%	1	1	0.04%

### Q1. Were there associations between reptile captures and vegetation type, did species detections change over time, and do trajectories differ between vegetation types?

**Fig 2:**
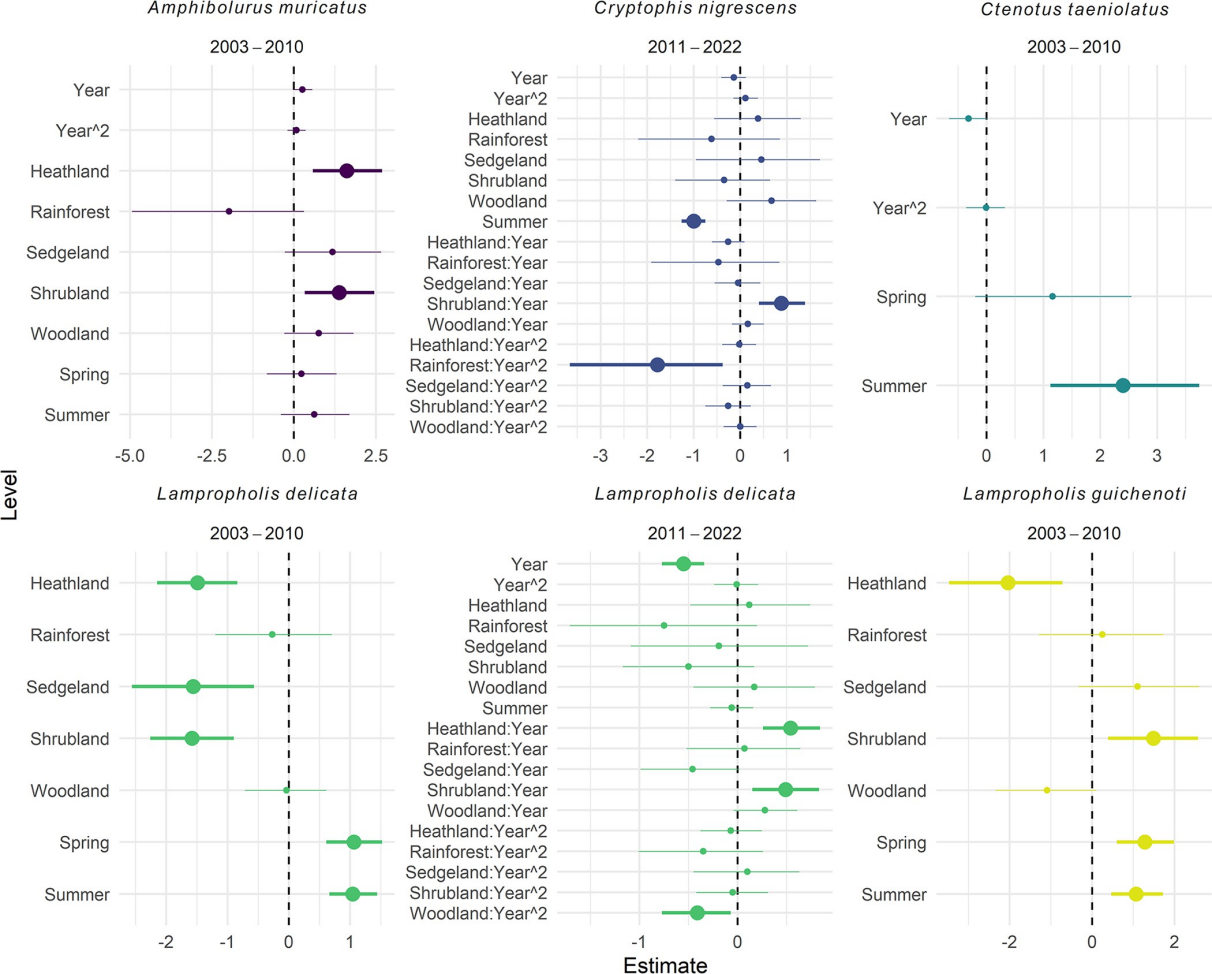
Effect sizes (posterior estimates) for the best-fit models testing the response of detection rate in vegetation types or different trajectories over time within vegetation types (see S4 Table in S1 File for model selection results and S5 Table in S1 File for posterior model estimates tables). Vegetation types (heathland, rainforest, sedgeland, shrubland, and woodland) are compared to forest. Spring and Summer are compared to Autumn. Error bars represent 95% credible intervals. We considered effects ‘significant’ if their 95% credible intervals did not cross the zero-effect line (larger points).

**Fig 3:**
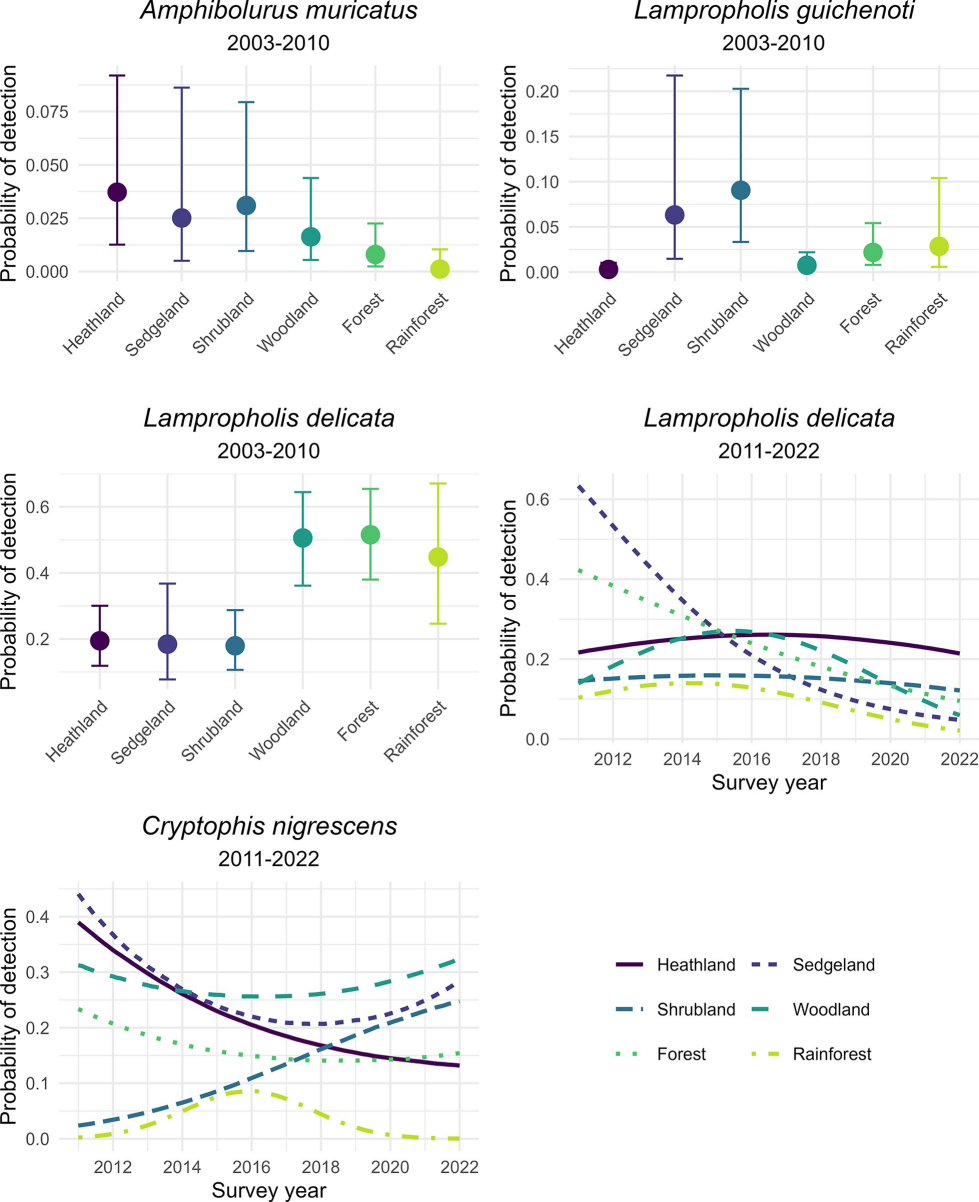
Predicted plots of species that exhibited a change in detection rate between vegetation types or different trajectories over time within vegetation types. Error bars represent 95% credible intervals. Only those effects considered significant (Fig 2) are plotted. See S5 Table in S1 File for posterior model estimates tables.

Four species exhibited responses to vegetation type or an interaction between vegetation type and time (Figs 2 and 3 and S4 Table in S1 File). From 2003 to 2010, *A*. *muricatus* was detected more frequently in heathland, sedgeland, and shrubland compared with forest and rainforest. Similarly, *L*. *guichenoti* was detected more frequently in sedgeland and shrubland than in other vegetation types, but this pattern occurred only in the early sampling period (Figs 2 and 3). In contrast, *L*. *delicata* was detected more frequently in woodland, forest, and rainforest from 2003 to 2011, but there were declines in detections in forest and sedgeland from 2011 to 2022 (Fig 3). *C*. *nigrescens* exhibited differing trajectories over time from 2011 to 2022, with decreases in heathland and forest over time (Fig 2).

### Q2. Did fire and differences in climate during the study period influence reptile captures?

**Fig 4:**
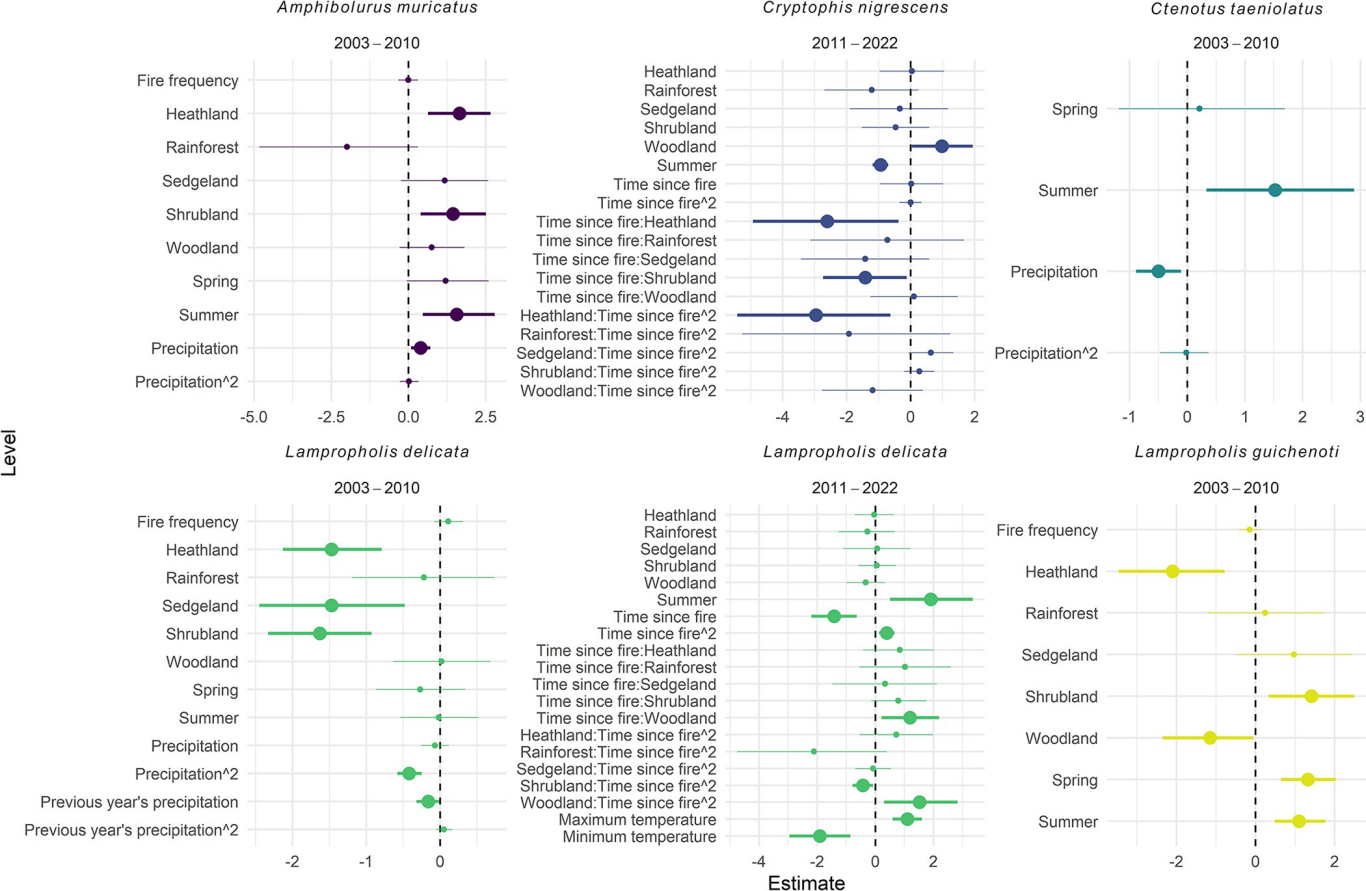
Effect sizes (posterior estimates) for the best-fit models testing the response of detection rate to the fire and climate variables. Vegetation types (heathland, rainforest, sedgeland, shrubland, and woodland) are compared to forest. Spring and Summer are compared to Autumn. Error bars represent 95% credible intervals. We considered effects ‘significant’ if their 95% credible intervals did not cross the zero-effect line (larger points). (see S6 Table in S1 File for model selection results and S7 Table in S1 File for posterior model estimates tables).

**Fig 5:**
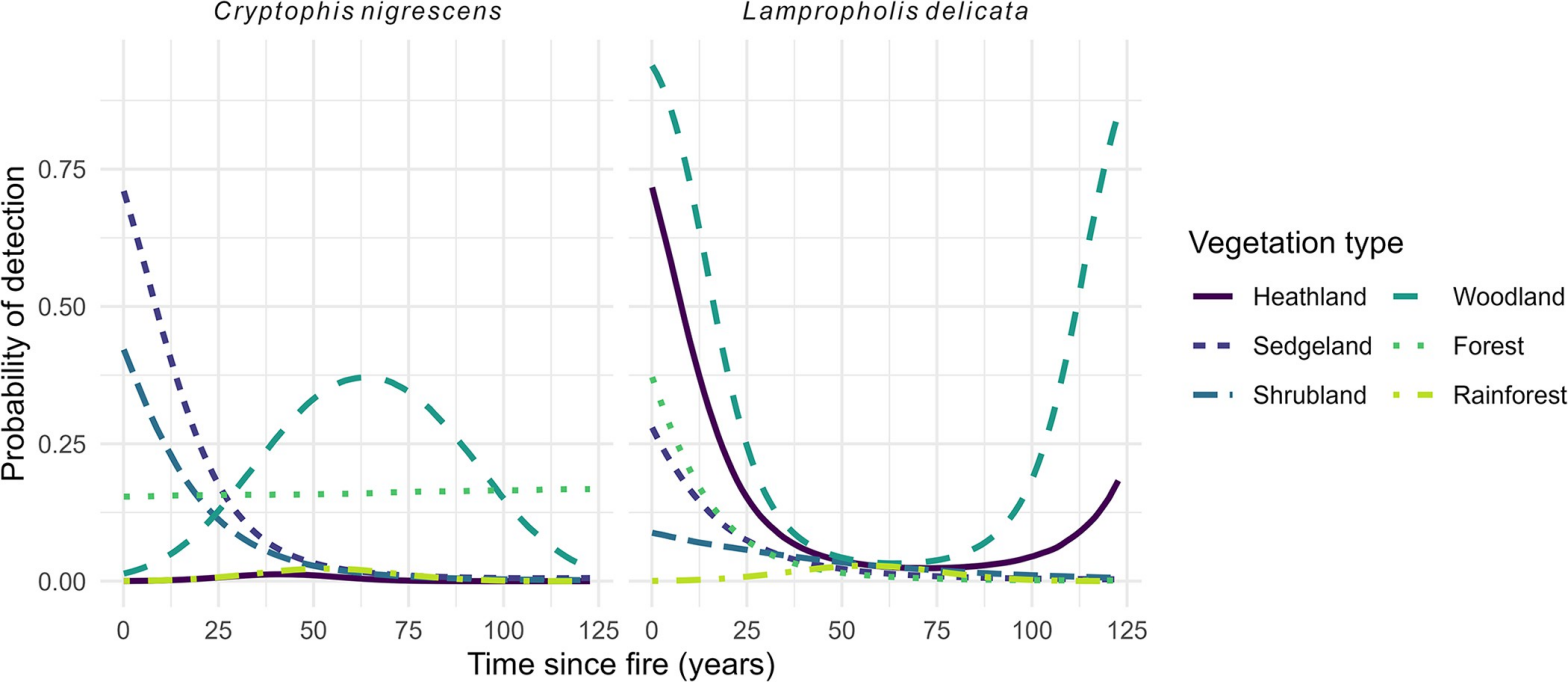
*C*. *nigrescens* and *L*. *delicata* responses to time since fire and vegetation variables for the 2011–2022 time period. See S7 Table in S1 File for posterior model estimates tables.

The only species to exhibit responses to fire variables were *C*. *nigrescens* and *L*. *delicata* in the later time period (Figs 4 and 5, S6 and S7 Tables in S1 File). Both species demonstrated interactive effects between time since fire and vegetation type (Figs 4 and 5). Fire frequency was a variable in the top-ranked models for *A*. *muricatus*, *L*. *delicata* (2003–2010), and *L*. *guichenoti*, however, the model estimates were not significant (Fig 4).

**Fig 6:**
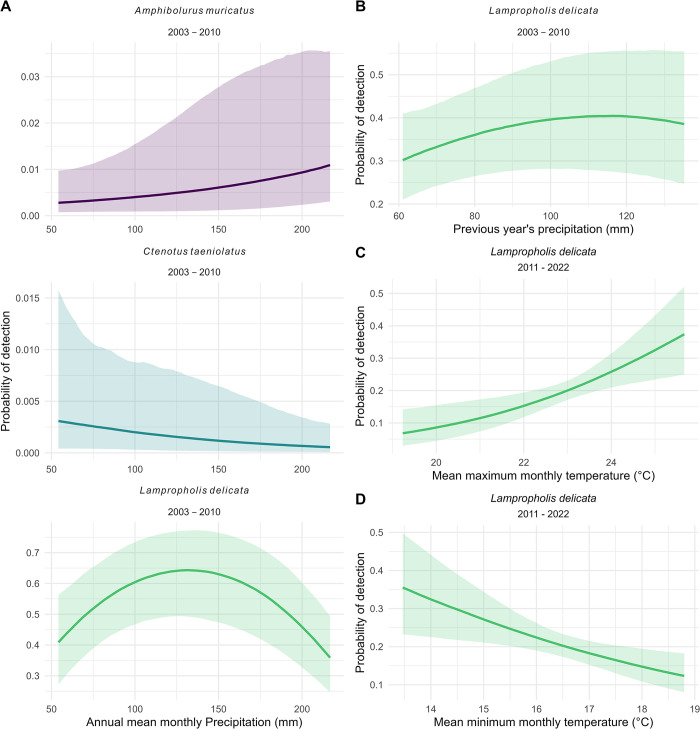
Effect sizes (posterior estimates) for the best-fit models testing the response of detection rate to the climate variables. A. Predicted plots of *A*. *muricatus*, *C*. *taeniolatus*, and *L*. *delicata* responses to annual mean monthly precipitation for the 2003–2010 time period. B. Predicted plot of *L*. *delicata* responses to previous year’s precipitation for the 2003–2010 time period. C. Predicted plot of *L*. *delicata* responses to mean maximum monthly temperature for the 2011–2022 time period. D. Predicted plot of *L*. *delicata* responses to mean minimum monthly temperature for the 2011–2022 time period. Only those effects considered significant are plotted. Error bands are 95% credible intervals. (see S6 Table in S1 File for model selection results and S7 Table in S1 File for posterior model estimates tables).

Three species were characterized by changing probabilities of detection from 2003 to 2010 in response to increased precipitation (*A*. *muricatus*, *C*. *taeniolatus*, and *L*. *delicata*) (Fig 6, S7 Table in S1 File). *L*. *delicata* exhibited an increase in probability of detection when precipitation was higher in the previous year (Fig 6B). This species also demonstrated a higher chance of detection when maximum temperatures were higher and minumim temperatures were lower (Fig 6C and 6D).

### Q3. Did the change in survey methodology breach the integrity of our long-term data?

We were able to analyse data for only *L*. *delicata* over both time periods. The inclusion of the *M*_*t*_ variable significantly improved every model fit (S8 Table in S1 File). Further, the top-ranked model included the interactions of vegetation type, time, and survey methodology. However, despite this model being the most parsimonious fit, the majority of the effects were not significant (S3 Fig and S9 Table in S1 File). The only significant effects were a difference in detections in Spring and Summer compared to Autumn, and a significant negative effect of the later time period (2011–2022) relative to the earlier time period (2003–2010). That is, that detections were much less likely in the second data collection surveys (based on substrates) than the first data collection surveys (which employed pitfall traps).

## Discussion

Long-term data, such as those presented here, are rare for reptiles in Australia [67]. Analyses of our data revealed several important aspects of reptile dynamics in response to multiple disturbances. We were able to analyse only one species across both time periods in our 19-year study. Further, the results for this species were inconsistent, leaving the question of whether the change in results between the two time periods was driven by changes over time, between survey methods, or a combination of both these factors. Our results were tempered by very low detections of almost all species for both of our study periods. Nevertheless, we identified clear associations of species with certain vegetation types, responses to climate, and, for one species, a strong association with fire. In the remainder of this paper, we further discuss these responses, followed by a deeper exploration of the implications of changing data collection methods for detection rates and possible ways to overcome its associated challenges.

It is important, from the outset, to acknowledge that reptile detections are a reflection of both whether the reptile occupies a given site, and whether it is possible to detect an individual in a given survey. The ability to detect a species in any given survey, therefore, could depend on a number of factors that change between surveys. Reptiles are ectothermic and their behaviour is influenced by weather. Therefore, a key factor influencing detection might be temperature and rainfall. Hence, any interpretation of field survey results should be mindful of how any relevant variable might influence occupancy and detection. For example, we found that three species showed a significant response to seasonal precipitation (as discussed further below).

We found only limited evidence of overall long-term changes in detections of the species we examined. Our data showed that detections of *L*. *delicata* declined from around 50% to around 10% probability of capture from 2011 to 2022 in sedgeland and forest vegetation types, and declined from about 10% to less than 5% in rainforest. This species is associated with forests, rainforests, and woodlands [52, 68], as our results from 2003–2010 confirmed. This decline is surprising given that this species is an extremely common and adaptable species, including being an invasive animal in New Zealand, Hawaii, and on Lord Howe Island [69–71].

The models for several species included associations with broad vegetation types. We recorded more detections of *L*. *delicata* in habitats with greater overstorey (woodland, forest, rainforest) than other vegetation types (heathland, sedgeland, shrubland). This species is generally associated with forest habitats [36, 37]. Interestingly, this pattern was reversed for *L*. *guichenoti* which was more often associated with sedgeland and shrubland than other habitat types. *L*. *guichenoti* is a generalist species often found in backyard gardens and urban areas [38]. It was shown to recover from drought more readily than *L*. *delicata* [72]. *L*. *guichenoti*, therefore, is likely better suited to the habitat types with less overstorey canopy than *L*. *delicata*, an interpretation broadly consistent with our results.

We found that detections of *C*. *nigrescens* were associated with fire, particularly in sedgeland and shrubland, where the species was less likely to be detected with increasing time since fire. This result contrasts with the findings of an earlier investigation which showed that *C*. *nigrescens* abundance decreased following wildfire [73]. We also found similar results for *L*. *delicata*, which was less likely to be detected with increasing time since fire until around 75 years following fire, when its detection probability increased. Please note that only sedgeland, shrubland, and forest vegetation types contained sites where the last fire was over 50 years ago (S4 Fig in S1 File). Again, this contrasts with previous research that suggests that *L*. *delicata* is slow to recover from fire. We therefore interpret both species’ short-term responses as potentially driven by the reduction of vegetation and litter following fire leading to these species using our artificial substrates for shelter, basking, and foraging [44, 74]. The increase in the long term for *L*. *delicata*, therefore, could be a reflection of their recovery over the long term in the surrounding habitat, whereas the short-term increase could be a reflection of this species using the substrates for habitat.

Increased precipitation resulted in contrasting responses for each species. Detections of *C*. *taeniolatus* decreased with increased precipitation, whereas detections of *A*. *muricatus* increased. Detections of *L*. *delicata* demonstrated a unimodal relationship with precipitation. The previous year’s precipitation was correlated with increased detections of *L*. *delicata*. Precipitation varied considerably during our study, with increases in precipitation towards the ends of both time periods (S1 Fig in S1 File). Indeed, the end of the first survey period (2002–2010) coincided with the breaking of the Millenium Drought [35], and with it, a period of wetter weather. Spence-Bailey et al. [50] in a study in the Australian semi-arid mallee region, recorded fewer detections of some species of reptiles during humid conditions and on overcast days. They discussed how wet conditions were often accompanied by lower temperatures, in which reptiles would be less active [50]. However, Ryan et al. [75], discovered that reptiles in the Los Pino Mountains in New Mexico, US, preferred foraging in sunny microhabitats following rainfall events and shaded habitats during dry periods. Further, increased precipitation is also thought to increase reproduction by some reptiles [59]. Whilst our study contrasts in both habitat conditions and species identities to those mentioned above, our results likely point to species-specific responses to rain, as well the difficulties in disentangling the effects of occupancy and activity on reptile detections.

The responses of *L*. *delicata* to mean maximum and minimum monthly temperatures are difficult to explain. These results could be another example of a combination of activity- and occupancy-related effects. The increase of detection when minimum temperatures are colder could be as a result of this species using the artificial substrates for their thermal properties, as found with other species of reptiles in Southern California [76]. In contrast, the increase in detection when maximum temperatures are warmer could be as a result of their increased activity in warmer temperatures.

Our results were strongly influenced by the change in sampling regime in 2011. This highlights the importance of structured and consistent long-term data collection. As Lindenmayer et al. [77] stated in Rule 5 of their ‘Eight things you should never do in a monitoring program’:

*‘Never change the way you monitor something without ensuring new methods can be calibrated with the old ones.’ [77]*.

Lindenmayer et al. [77] cite a controversial case involving levels of silicon in Lake Michigan in the USA as an example [78]. In this case, major decreases in apparent silicon levels in Lake Michigan coincided with a change in the laboratory analysing the water samples for the data. This change completely confounded the true trajectory of silicon levels in the lake with a change of data collection methodology. The actual ‘true’ trajectory is still unknown.

Taken at face value, our results might indicate that there has been a sudden increase in *C*. *nigrescens* from 2011 onwards (six detections prior, 634 after 2011, Table 4). Likewise, they could indicate a sudden decrease in *L*. *guichenoti* (301 prior, 29 after) and *A*. *muricatus* (123 prior, six after). However, in our case, a change in survey method was confounded with time, making it difficult to determine whether the effect was due to a change in methodology, a change over time, or a combination of the two.

Rule 5 in Lindenmayer et al. [77], while clearly broken in our case, provides calibration as a solution for researchers needing to change their field methodology following several years of established data collection. Calibration of all or a subset of a sampling regime can maintain the integrity of the long-term data by establishing a period of time in which both the old and new methodologies are conducted simultaneously. This crossover period then allows for any differences in output between the two periods to be accounted for in subsequent analyses [77]. In hindsight, therefore, a period of calibration at several of our sites and over a period of time would have mitigated the impact of the survey change on our data. The resultant data analysis would, therefore, have allowed us to model the effects of both time and survey method with confidence that they were not confounded. With this in mind, we plan to reintroduce the original surveying methodology in a subset of sites at BNP over several sampling seasons. We hope to be able to use the data collection to calibrate the original long-term data.

Unfortunately, in our case, calibration would have been useful for only one of the species we detected (*L*. *delicata*). Indeed, a critical factor in our study was that our survey methods enabled the collection of sufficient data for meaningful statistical analysis for a small subset of reptile species. Many species of reptiles are notoriously difficult to detect. For example, the skink *Lampropholis elongata*, endemic to the New England region of New South Wales, Australia, was not seen for nine years until a concerted effort to detect the species was conducted [79]. Furthermore, the species was undetected in pitfall trapping surveys, being recorded only in active searches [79]. *L*. *delicata* is known to be a very active species, much more than the congeneric *L*. *guichenoti* [80], which likely contributes to its high detection rate compared to other species.

Several studies have found that one of the most effective survey methods for reptiles is active searching, which can be complemented with the deployment of artificial substrates and/or pitfall traps [22, 51]. These techniques are very resource intensive in terms of infrastructure, time, and expertise. Indeed, the extensive surveys undertaken for 19 years at BNP were a significant logistical and financial investment, as was the time in pursuing and gathering the funding to maintain the data collection. Funding for research and monitoring is notoriously difficult to attain and almost always awarded for short-term time periods [7], and therefore not well suited to long-term data collection. This creates a ‘wicked problem’ where data to monitor populations effectively requires consistent and resource-intensive surveying, yet the resources required for this type of surveying rely on funding which are very difficult to attain. Overcoming this wicked problem is one of the greatest challenges for the monitoring of biodiversity [8].

## Conclusions

Our study revealed declines in detections of two skink species over time (*L*. *delicata*, *C*. *taeniolatus*), which we suspect was partly driven by milder weather influencing activity levels of these species. Our study also identified broad vegetation type associations of two congeneric species. *L*. *delicata* was associated with forested sites with high overstorey, and *L*. *guichenoti* was associated with more shrubby sites with comparatively lower overstorey. Our results also contained evidence of associations between *C*. *nigrescens* and *L*. *delicata* and time since fire. However, our results were strongly influenced by a change in sampling regime that may have breached the integrity of the long-term dataset. Further, we failed to undertake a calibration study to allow us to account for the confounding of time and survey method. Researchers and environmental managers experience significant resource constraints. It is inevitable, therefore, that data collection will be subject to cost cutting in order to try to streamline the process. Whilst improvements in efficiencies might be possible with new technologies, it is very important to maintain the integrity of long-term datasets as data collection continues. A simple but crucial step that maintains the integrity of long-term datasets is to conduct calibration that allows subsequent analysis to control for a change in surveying methodology.

## Supporting information

S1 File (DOCX)
