# Supplementary material for: A misleading tail: A long-term study of reptile responses to multiple disturbances undermined by a change in surveying techniques
Source: PLoS One. 2024 Jun 14;19(6):e0305518. doi: 10.1371/journal.pone.0305518 (PMC11178227; doi:10.1371/journal.pone.0305518)
Supplement: S1 File — (DOCX) [file pone.0305518.s001.docx]

Supplementary materials

A misleading tail: a long-term study of reptile responses to multiple disturbances undermined by a change in surveying techniques

Maldwyn John Evans**^1^ ,** Christopher MacGregor**^1^**, and David Lindenmayer**^1^**

**^1^**Fenner School of Environment and Society, The Australian National University, Canberra, ACT, 2601, Australia

**Table S1. Pitfall trap sizes used at each site from 2003 until 2010.**

| Pitfall Trap Number | Diameter (mm) | Depth (mm) |
| --- | --- | --- |
| 1 | 250 | 250 |
| 2 | 300 | 300 |
| 3 | 300 | 400 |
| 4 | 300 | 300 |
| 5 | 300 | 400 |
| 6 | 300 | 300 |

**Table S2. Number of surveys undertaken in each month in each year from 2003 until 2010.**

| Month | 2003 | 2004 | 2005 | 2006 | 2007 | 2008 | 2009 | 2010 |
| --- | --- | --- | --- | --- | --- | --- | --- | --- |
| 1 | 0 | 0 | 83 | 81 | 3 | 17 | 21 | 0 |
| 2 | 0 | 88 | 74 | 96 | 86 | 67 | 60 | 85 |
| 3 | 0 | 0 | 5 | 0 | 3 | 7 | 3 | 0 |
| 4 | 61 | 51 | 0 | 0 | 0 | 0 | 0 | 0 |
| 5 | 51 | 0 | 0 | 0 | 0 | 0 | 0 | 0 |
| 9 | 0 | 0 | 0 | 0 | 0 | 0 | 7 | 0 |
| 10 | 0 | 0 | 0 | 0 | 0 | 0 | 37 | 0 |
| 11 | 0 | 98 | 62 | 0 | 61 | 0 | 36 | 0 |
| 12 | 0 | 0 | 1 | 64 | 14 | 102 | 42 | 16 |

**Table S3. Number of site surveys undertaken in each season in each year from 2003 until 2010.**

| Month | 2003 | 2004 | 2005 | 2006 | 2007 | 2008 | 2009 | 2010 |
| --- | --- | --- | --- | --- | --- | --- | --- | --- |
| Autumn | 112 | 51 | 5 | 0 | 3 | 7 | 3 | 0 |
| Spring | 0 | 98 | 62 | 0 | 61 | 0 | 80 | 0 |
| Summer | 0 | 88 | 158 | 241 | 103 | 186 | 123 | 101 |

**Table S4. Model selection results for Question 1. Y=Year, V= Vegetation, V:Y=Vegetation * year interaction. Models <2 ΔLOOIC are shown.**

| Species | Period | Y | V | V:Y | LOOIC | ΔLOOIC |
| --- | --- | --- | --- | --- | --- | --- |
| *Amphibolurus muricatus* | 03-10 | ✓ | ✓ |  | 676.25 | 0.00 |
| *Cryptophis nigrescens* | 11-22 | ✓ | ✓ | ✓ | 1,853.74 | 0.00 |
| *Ctenotus taeniolatus* | 03-10 | ✓ |  |  | 475.76 | 0.00 |
|  | 03-10 | ✓ | ✓ |  | 475.93 | 0.17 |
| *Lampropholis delicata* | 03-10 |  | ✓ |  | 1,725.47 | 0.00 |
|  | 11-22 | ✓ | ✓ | ✓ | 2,444.13 | 0.00 |
| *Lampropholis guichenoti* | 03-10 |  | ✓ |  | 879.90 | 0.00 |
|  | 03-10 | ✓ | ✓ |  | 880.54 | 0.64 |

**Table S5. Posterior model estimates for Question 1. SE = standard error, ESS = effective sample size.**

| Species | Period | Level | Estimate | SE | Lower | Upper | Rhat | Bulk ESS | Tail ESS |
| --- | --- | --- | --- | --- | --- | --- | --- | --- | --- |
| *Amphibolurus muricatus* | 03-10 | Intercept | -4.74 | 0.70 | -6.17 | -3.41 | 1.00 | 2490.75 | 2903.48 |
|  |  | Year | 0.26 | 0.15 | -0.03 | 0.56 | 1.00 | 3605.24 | 3046.09 |
|  |  | Year^2 | 0.08 | 0.14 | -0.20 | 0.36 | 1.00 | 3789.14 | 3188.83 |
|  |  | Heathland | 1.61 | 0.54 | 0.58 | 2.69 | 1.00 | 2068.36 | 2599.99 |
|  |  | Rainforest | -1.98 | 1.33 | -4.94 | 0.31 | 1.00 | 5510.87 | 2573.68 |
|  |  | Sedgeland | 1.18 | 0.75 | -0.28 | 2.66 | 1.00 | 2247.83 | 2744.92 |
|  |  | Shrubland | 1.38 | 0.55 | 0.33 | 2.45 | 1.00 | 2022.76 | 2880.89 |
|  |  | Woodland | 0.76 | 0.54 | -0.29 | 1.82 | 1.00 | 2029.94 | 2492.40 |
|  |  | Spring | 0.23 | 0.54 | -0.83 | 1.30 | 1.00 | 3380.56 | 3115.20 |
|  |  | Summer | 0.62 | 0.54 | -0.40 | 1.70 | 1.00 | 2892.73 | 2849.66 |
| *Cryptophis nigrescens* | 11-22 | Intercept | -1.74 | 0.35 | -2.42 | -1.04 | 1.00 | 749.86 | 1460.59 |
|  |  | Heathland | 0.38 | 0.48 | -0.56 | 1.30 | 1.00 | 829.74 | 1453.52 |
|  |  | Rainforest | -0.62 | 0.78 | -2.19 | 0.85 | 1.00 | 1921.58 | 2530.16 |
|  |  | Sedgeland | 0.45 | 0.67 | -0.95 | 1.71 | 1.01 | 872.34 | 1631.31 |
|  |  | Shrubland | -0.35 | 0.52 | -1.40 | 0.64 | 1.00 | 1004.84 | 1643.64 |
|  |  | Woodland | 0.67 | 0.50 | -0.29 | 1.63 | 1.01 | 731.93 | 1634.41 |
|  |  | Year | -0.14 | 0.14 | -0.41 | 0.12 | 1.00 | 1538.00 | 2507.88 |
|  |  | Year^2 | 0.11 | 0.14 | -0.15 | 0.38 | 1.00 | 1834.06 | 2181.46 |
|  |  | Summer | -1.00 | 0.13 | -1.26 | -0.75 | 1.00 | 5536.53 | 3215.59 |
|  |  | Heathland:Year | -0.26 | 0.18 | -0.61 | 0.09 | 1.00 | 2207.17 | 2962.48 |
|  |  | Rainforest:Year | -0.47 | 0.70 | -1.91 | 0.84 | 1.00 | 4607.49 | 2717.84 |
|  |  | Sedgeland:Year | -0.05 | 0.25 | -0.55 | 0.43 | 1.00 | 2950.69 | 2873.67 |
|  |  | Shrubland:Year | 0.88 | 0.26 | 0.40 | 1.39 | 1.00 | 2443.32 | 2790.48 |
|  |  | Woodland:Year | 0.16 | 0.18 | -0.18 | 0.51 | 1.00 | 2099.08 | 3075.75 |
|  |  | Heathland:Year^2 | -0.02 | 0.19 | -0.39 | 0.34 | 1.00 | 2527.59 | 2673.38 |
|  |  | Rainforest:Year^2 | -1.78 | 0.84 | -3.66 | -0.38 | 1.00 | 3714.69 | 2560.49 |
|  |  | Sedgeland:Year^2 | 0.15 | 0.27 | -0.38 | 0.66 | 1.00 | 3575.47 | 3347.17 |
|  |  | Shrubland:Year^2 | -0.26 | 0.24 | -0.75 | 0.23 | 1.00 | 2675.91 | 2485.85 |
|  |  | Woodland:Year^2 | 0.00 | 0.18 | -0.36 | 0.35 | 1.00 | 2190.20 | 2644.78 |
| *Ctenotus taeniolatus* | 03-10 | Intercept | -6.99 | 0.94 | -8.95 | -5.22 | 1.00 | 2305.26 | 2338.74 |
|  |  | Year | -0.32 | 0.17 | -0.66 | 0.01 | 1.00 | 4791.65 | 3178.10 |
|  |  | Year^2 | -0.01 | 0.17 | -0.36 | 0.32 | 1.00 | 4449.42 | 3173.86 |
|  |  | Spring | 1.16 | 0.71 | -0.20 | 2.55 | 1.00 | 3773.53 | 3159.98 |
|  |  | Summer | 2.40 | 0.68 | 1.12 | 3.74 | 1.00 | 3381.95 | 3141.20 |
| *Lampropholis delicata* |  | Intercept | 0.06 | 0.29 | -0.49 | 0.64 | 1.00 | 1771.58 | 2571.20 |
|  |  | Heathland | -1.49 | 0.34 | -2.15 | -0.84 | 1.00 | 1897.69 | 2696.91 |
|  |  | Rainforest | -0.27 | 0.48 | -1.20 | 0.70 | 1.00 | 2231.26 | 2958.31 |
|  |  | Sedgeland | -1.56 | 0.51 | -2.56 | -0.57 | 1.00 | 2102.95 | 2666.25 |
|  |  | Shrubland | -1.58 | 0.35 | -2.26 | -0.90 | 1.00 | 2094.49 | 2343.83 |
|  |  | Woodland | -0.04 | 0.34 | -0.72 | 0.61 | 1.00 | 1599.99 | 2266.03 |
|  |  | Spring | 1.06 | 0.23 | 0.61 | 1.52 | 1.00 | 4315.74 | 3092.47 |
|  |  | Summer | 1.04 | 0.20 | 0.66 | 1.44 | 1.00 | 4578.46 | 3272.70 |
|  | 11-22 | Intercept | -1.17 | 0.23 | -1.64 | -0.74 | 1.00 | 3008.89 | 4037.12 |
|  |  | Heathland | 0.12 | 0.31 | -0.48 | 0.74 | 1.00 | 2967.50 | 4237.65 |
|  |  | Rainforest | -0.75 | 0.48 | -1.71 | 0.20 | 1.00 | 4894.99 | 5930.12 |
|  |  | Sedgeland | -0.19 | 0.46 | -1.09 | 0.72 | 1.00 | 4328.13 | 5055.67 |
|  |  | Shrubland | -0.50 | 0.34 | -1.17 | 0.17 | 1.00 | 3662.98 | 5288.34 |
|  |  | Woodland | 0.17 | 0.32 | -0.45 | 0.79 | 1.00 | 3121.60 | 4801.99 |
|  |  | Year | -0.55 | 0.11 | -0.77 | -0.34 | 1.00 | 4292.20 | 5078.31 |
|  |  | Year^2 | -0.01 | 0.11 | -0.24 | 0.21 | 1.00 | 4438.56 | 5472.50 |
|  |  | Summer | -0.06 | 0.11 | -0.28 | 0.16 | 1.00 | 11216.56 | 6279.53 |
|  |  | Heathland:Year | 0.54 | 0.15 | 0.26 | 0.84 | 1.00 | 5207.21 | 5186.83 |
|  |  | Rainforest:Year | 0.07 | 0.30 | -0.52 | 0.64 | 1.00 | 8430.69 | 5774.24 |
|  |  | Sedgeland:Year | -0.46 | 0.26 | -0.99 | 0.02 | 1.00 | 7958.67 | 6024.52 |
|  |  | Shrubland:Year | 0.49 | 0.17 | 0.15 | 0.83 | 1.00 | 5931.40 | 6097.23 |
|  |  | Woodland:Year | 0.28 | 0.17 | -0.04 | 0.61 | 1.00 | 5897.49 | 6056.12 |
|  |  | Heathland:Year^2 | -0.07 | 0.16 | -0.38 | 0.25 | 1.00 | 5590.02 | 6466.21 |
|  |  | Rainforest:Year^2 | -0.35 | 0.32 | -1.01 | 0.26 | 1.00 | 7837.55 | 6229.03 |
|  |  | Sedgeland:Year^2 | 0.10 | 0.27 | -0.45 | 0.63 | 1.00 | 7281.67 | 5750.43 |
|  |  | Shrubland:Year^2 | -0.05 | 0.19 | -0.42 | 0.31 | 1.00 | 5990.26 | 6158.95 |
|  |  | Woodland:Year^2 | -0.41 | 0.18 | -0.77 | -0.07 | 1.00 | 5825.39 | 5838.64 |
| *Lampropholis guichenoti* | 03-10 | Intercept | -3.81 | 0.50 | -4.82 | -2.86 | 1.00 | 2980.67 | 5256.26 |
|  |  | Heathland | -2.04 | 0.69 | -3.47 | -0.72 | 1.00 | 4055.56 | 5629.42 |
|  |  | Rainforest | 0.25 | 0.77 | -1.29 | 1.73 | 1.00 | 3608.34 | 4603.60 |
|  |  | Sedgeland | 1.10 | 0.75 | -0.33 | 2.60 | 1.00 | 2932.45 | 4417.25 |
|  |  | Shrubland | 1.49 | 0.55 | 0.39 | 2.57 | 1.00 | 2153.80 | 3602.88 |
|  |  | Woodland | -1.09 | 0.61 | -2.34 | 0.10 | 1.00 | 3063.44 | 4595.55 |
|  |  | Spring | 1.28 | 0.35 | 0.60 | 1.99 | 1.00 | 6433.03 | 6076.81 |
|  |  | Summer | 1.07 | 0.32 | 0.46 | 1.72 | 1.00 | 6308.81 | 6107.18 |

**Table S6. Model selection results for Question 2.** V= Vegetation, FP= Fire frequency, FT=Time since fire, Tmax=Mean maximum monthly temperature, Tmin=Mean minimum monthly temperature, P=Annual precipitation, PPR= Annual precipitation in previous year, “:” indicates an interaction term. Models <2 ΔLOOIC are shown.

| Species | Period | V | FP | FT | FT:V | FP:V | Tmax | Tmin | P | PPR | LOOIC | ΔLOOIC |
| --- | --- | --- | --- | --- | --- | --- | --- | --- | --- | --- | --- | --- |
| *Amphibolurus muricatus* | 03-10 |  |  |  |  |  | ✓ |  | ✓ |  | 677.00 | 0.00 |
|  | 03-10 |  |  |  |  |  |  |  | ✓ |  | 677.05 | 0.05 |
|  | 03-10 |  |  |  |  |  |  | ✓ | ✓ |  | 677.46 | 0.46 |
|  | 03-10 |  |  |  |  |  |  | ✓ | ✓ | ✓ | 678.48 | 1.48 |
|  | 03-10 |  |  |  |  |  | ✓ | ✓ | ✓ |  | 678.71 | 1.71 |
|  | 03-10 | ✓ | ✓ |  |  |  |  |  |  |  | 678.82 | 1.82 |
| *Cryptophis nigrescens* | 11-22 | ✓ |  | ✓ | ✓ |  |  |  |  |  | 1,840.98 | 0.00 |
| *Ctenotus taeniolatus* | 03-10 |  |  |  |  |  |  |  | ✓ |  | 471.39 | 0.00 |
|  | 03-10 |  |  |  |  |  |  | ✓ | ✓ |  | 472.27 | 0.88 |
|  | 03-10 |  |  |  |  |  | ✓ |  | ✓ |  | 472.48 | 1.09 |
| *Lampropholis delicata* | 03-10 |  |  |  |  |  |  | ✓ | ✓ | ✓ | 1,693.44 | 0.00 |
|  | 03-10 |  |  |  |  |  | ✓ |  | ✓ | ✓ | 1,693.57 | 0.13 |
|  | 03-10 |  |  |  |  |  |  |  | ✓ | ✓ | 1,695.04 | 1.60 |
|  | 11-22 | ✓ |  | ✓ | ✓ |  |  |  |  |  | 2,469.04 | 0.00 |
| *Lampropholis guichenoti* | 03-10 | ✓ | ✓ |  |  |  |  |  |  |  | 881.58 | 0.00 |
|  | 03-10 | ✓ | ✓ |  |  | ✓ |  |  |  |  | 881.65 | 0.07 |

**Table S7. Posterior model estimates for Question 2.** SE = standard error, ESS = effective sample size.

| Species | Period | Level | Estimate | SE | Lower | Upper | Rhat | Bulk ESS | Tail ESS |
| --- | --- | --- | --- | --- | --- | --- | --- | --- | --- |
| *Amphibolurus muricatus* | 2003-2010 | Intercept | -5.51 | 0.79 | -7.12 | -4.04 | 1.00 | 4489.10 | 4265.53 |
|  |  | Fire frequency | -0.01 | 0.16 | -0.33 | 0.31 | 1.00 | 6402.08 | 5494.58 |
|  |  | Heathland | 1.65 | 0.52 | 0.63 | 2.66 | 1.00 | 4233.28 | 5577.52 |
|  |  | Rainforest | -1.99 | 1.32 | -4.83 | 0.31 | 1.00 | 10535.63 | 5681.00 |
|  |  | Sedgeland | 1.17 | 0.72 | -0.25 | 2.58 | 1.00 | 5516.77 | 6053.62 |
|  |  | Shrubland | 1.44 | 0.54 | 0.39 | 2.51 | 1.00 | 4030.77 | 4475.19 |
|  |  | Woodland | 0.75 | 0.54 | -0.29 | 1.81 | 1.00 | 4529.09 | 4972.41 |
|  |  | Spring | 1.20 | 0.68 | -0.06 | 2.59 | 1.00 | 6063.00 | 5561.01 |
|  |  | Summer | 1.56 | 0.60 | 0.46 | 2.79 | 1.00 | 5603.95 | 5253.99 |
|  |  | Precipitation | 0.39 | 0.16 | 0.08 | 0.71 | 1.00 | 9418.75 | 5935.01 |
|  |  | Precipitation^2 | 0.01 | 0.15 | -0.28 | 0.32 | 1.00 | 5675.27 | 5641.74 |
| *Cryptophis nigrescens* | 2011-2022 | Intercept | -1.69 | 0.36 | -2.40 | -0.99 | 1.00 | 1760.31 | 3058.18 |
|  |  | Time since fire | 0.02 | 0.50 | -0.96 | 1.02 | 1.00 | 1824.21 | 2900.05 |
|  |  | Heathland | 0.04 | 0.52 | -0.97 | 1.05 | 1.00 | 2208.49 | 4044.17 |
|  |  | Rainforest | -1.22 | 0.75 | -2.70 | 0.24 | 1.00 | 2802.08 | 4850.35 |
|  |  | Sedgeland | -0.34 | 0.79 | -1.90 | 1.17 | 1.00 | 2556.95 | 3848.75 |
|  |  | Shrubland | -0.47 | 0.53 | -1.53 | 0.58 | 1.00 | 1624.80 | 2436.50 |
|  |  | Woodland | 0.98 | 0.48 | 0.03 | 1.94 | 1.00 | 1542.00 | 2855.65 |
|  |  | Time since fire^2 | 0.00 | 0.18 | -0.35 | 0.34 | 1.00 | 1912.81 | 2893.59 |
|  |  | Summer | -0.94 | 0.12 | -1.19 | -0.70 | 1.00 | 11273.86 | 5867.83 |
|  |  | Time since fire:Heathland | -2.61 | 1.17 | -4.93 | -0.38 | 1.00 | 3435.74 | 4061.10 |
|  |  | Time since fire:Rainforest | -0.73 | 1.23 | -3.14 | 1.66 | 1.00 | 5601.74 | 5740.10 |
|  |  | Time since fire:Sedgeland | -1.42 | 1.02 | -3.43 | 0.58 | 1.00 | 3368.68 | 5063.05 |
|  |  | Time since fire:Shrubland | -1.42 | 0.67 | -2.74 | -0.12 | 1.00 | 2190.13 | 3152.39 |
|  |  | Time since fire:Woodland | 0.10 | 0.70 | -1.26 | 1.47 | 1.00 | 2451.13 | 4031.67 |
|  |  | Heathland:Time since fire^2 | -2.96 | 1.22 | -5.42 | -0.63 | 1.00 | 5249.25 | 4861.42 |
|  |  | Rainforest:Time since fire^2 | -1.93 | 1.66 | -5.27 | 1.24 | 1.00 | 8068.66 | 5984.90 |
|  |  | Sedgeland:Time since fire^2 | 0.63 | 0.35 | -0.06 | 1.34 | 1.00 | 3305.06 | 4733.22 |
|  |  | Shrubland:Time since fire^2 | 0.27 | 0.24 | -0.20 | 0.74 | 1.00 | 2274.38 | 3625.95 |
|  |  | Woodland:Time since fire^2 | -1.19 | 0.81 | -2.77 | 0.38 | 1.00 | 4449.71 | 6020.62 |
| *Ctenotus taeniolatus* | 2003-2010 | Intercept | -6.27 | 0.89 | -8.18 | -4.64 | 1.00 | 2122.96 | 2032.54 |
|  |  | Precipitation | -0.50 | 0.20 | -0.89 | -0.11 | 1.00 | 4512.44 | 2981.06 |
|  |  | Precipitation^2 | -0.02 | 0.21 | -0.47 | 0.37 | 1.00 | 4214.31 | 3127.68 |
|  |  | Spring | 0.21 | 0.73 | -1.19 | 1.69 | 1.00 | 3783.01 | 2658.67 |
|  |  | Summer | 1.52 | 0.64 | 0.33 | 2.89 | 1.00 | 3568.09 | 2772.00 |
| *Lampropholis delicata* |  | Intercept | 1.38 | 0.37 | 0.65 | 2.11 | 1.00 | 2077.13 | 2904.04 |
|  |  | Fire frequency | 0.11 | 0.10 | -0.08 | 0.31 | 1.00 | 2665.53 | 3078.23 |
|  |  | Heathland | -1.47 | 0.34 | -2.13 | -0.79 | 1.00 | 1829.70 | 2615.82 |
|  |  | Rainforest | -0.22 | 0.49 | -1.19 | 0.74 | 1.00 | 2035.92 | 2289.86 |
|  |  | Sedgeland | -1.47 | 0.50 | -2.45 | -0.48 | 1.00 | 2139.99 | 2351.22 |
|  |  | Shrubland | -1.63 | 0.36 | -2.33 | -0.93 | 1.00 | 1618.67 | 2406.01 |
|  |  | Woodland | 0.02 | 0.33 | -0.64 | 0.68 | 1.00 | 1653.32 | 1967.05 |
|  |  | Precipitation | -0.07 | 0.10 | -0.26 | 0.12 | 1.00 | 5014.26 | 3239.61 |
|  |  | Precipitation^2 | -0.42 | 0.08 | -0.58 | -0.25 | 1.00 | 3474.53 | 3636.16 |
|  |  | Spring | -0.27 | 0.31 | -0.87 | 0.34 | 1.00 | 3474.86 | 3356.31 |
|  |  | Summer | -0.02 | 0.27 | -0.54 | 0.52 | 1.00 | 3439.67 | 2831.27 |
|  |  | Previous year's precipitation | -0.16 | 0.08 | -0.32 | -0.01 | 1.00 | 4591.31 | 3292.97 |
|  |  | Previous year's precipitation^2 | 0.05 | 0.05 | -0.05 | 0.17 | 1.00 | 5118.67 | 3348.90 |
|  | 2011-2022 | Intercept | -2.75 | 0.51 | -3.75 | -1.78 | 1.00 | 2347.02 | 2461.13 |
|  |  | Maximum temperature | 1.10 | 0.26 | 0.59 | 1.60 | 1.00 | 2831.87 | 2930.61 |
|  |  | Minimum temperature | -1.91 | 0.54 | -2.96 | -0.86 | 1.00 | 2471.89 | 2221.47 |
|  |  | Time since fire | -1.42 | 0.39 | -2.20 | -0.64 | 1.01 | 1035.84 | 1889.92 |
|  |  | Heathland | -0.04 | 0.34 | -0.71 | 0.64 | 1.00 | 1596.14 | 2455.87 |
|  |  | Rainforest | -0.27 | 0.49 | -1.26 | 0.67 | 1.00 | 2286.95 | 2209.37 |
|  |  | Sedgeland | 0.06 | 0.59 | -1.10 | 1.21 | 1.00 | 1824.95 | 2238.72 |
|  |  | Shrubland | 0.05 | 0.33 | -0.59 | 0.70 | 1.00 | 1398.91 | 2236.25 |
|  |  | Woodland | -0.33 | 0.34 | -1.00 | 0.34 | 1.00 | 1507.52 | 2098.44 |
|  |  | Time since fire^2 | 0.39 | 0.14 | 0.13 | 0.66 | 1.00 | 1177.62 | 1895.30 |
|  |  | Summer | 1.91 | 0.73 | 0.50 | 3.35 | 1.00 | 2771.12 | 2743.90 |
|  |  | Time since fire:Heathland | 0.84 | 0.62 | -0.42 | 2.03 | 1.01 | 1900.14 | 2653.48 |
|  |  | Time since fire:Rainforest | 1.02 | 0.80 | -0.55 | 2.60 | 1.00 | 2635.80 | 2986.37 |
|  |  | Time since fire:Sedgeland | 0.33 | 0.92 | -1.48 | 2.11 | 1.00 | 1742.33 | 2225.97 |
|  |  | Time since fire:Shrubland | 0.79 | 0.49 | -0.15 | 1.76 | 1.00 | 1176.63 | 2229.56 |
|  |  | Time since fire:Woodland | 1.19 | 0.50 | 0.21 | 2.19 | 1.00 | 1163.11 | 2127.53 |
|  |  | Heathland:Time since fire^2 | 0.72 | 0.65 | -0.55 | 1.98 | 1.00 | 3524.17 | 3207.09 |
|  |  | Rainforest:Time since fire^2 | -2.11 | 1.31 | -4.75 | 0.40 | 1.00 | 4268.41 | 3020.31 |
|  |  | Sedgeland:Time since fire^2 | -0.08 | 0.32 | -0.69 | 0.54 | 1.00 | 1734.35 | 2110.92 |
|  |  | Shrubland:Time since fire^2 | -0.43 | 0.18 | -0.78 | -0.08 | 1.00 | 1490.54 | 2003.66 |
|  |  | Woodland:Time since fire^2 | 1.52 | 0.63 | 0.30 | 2.83 | 1.00 | 3415.66 | 2705.99 |
| *Lampropholis guichenoti* | 2003-2010 | Intercept | -3.79 | 0.50 | -4.79 | -2.83 | 1.00 | 1745.39 | 2391.65 |
|  |  | Fire frequency | -0.15 | 0.15 | -0.42 | 0.14 | 1.00 | 2395.88 | 2934.49 |
|  |  | Heathland | -2.10 | 0.69 | -3.46 | -0.78 | 1.00 | 1889.74 | 2308.49 |
|  |  | Rainforest | 0.24 | 0.76 | -1.21 | 1.71 | 1.00 | 1928.32 | 2455.83 |
|  |  | Sedgeland | 0.97 | 0.74 | -0.49 | 2.45 | 1.00 | 1503.74 | 2031.82 |
|  |  | Shrubland | 1.41 | 0.54 | 0.33 | 2.49 | 1.00 | 1282.44 | 1997.12 |
|  |  | Woodland | -1.15 | 0.58 | -2.35 | -0.05 | 1.00 | 1453.45 | 1906.68 |
|  |  | Spring | 1.32 | 0.35 | 0.64 | 2.03 | 1.00 | 3058.61 | 3090.70 |
|  |  | Summer | 1.10 | 0.33 | 0.48 | 1.77 | 1.00 | 2962.76 | 2986.26 |

**Table S8. Model selection table for models using data from both time periods combined to test the effects of all predictors on *L. delicata* detections (Question 3).** Y=Year, V= Vegetation, Tmax=Mean maximum monthly temperature, Tmin=Mean minimum monthly temperature, P=Annual precipitation, PPR= Annual precipitation in previous year, M=time period/survey methodology, “:” indicates an interaction term.The top-ranked model includes the term for the interaction of vegetation, time, and time period (V:Y:TP). only the six highest-ranked models are shown.

| Y | V | V:Y | Tmax | Tmin | P | PPR | M | V:Y:M | LOOIC | ΔLOOIC |
| --- | --- | --- | --- | --- | --- | --- | --- | --- | --- | --- |
| *✓* | ✓ | ✓ |  |  |  |  | ✓ | ✓ | 4,309.02 | 0.00 |
| *✓* | ✓ | ✓ |  |  |  |  | ✓ |  | 4,319.36 | 10.34 |
| *✓* | ✓ | ✓ |  |  |  |  |  |  | 4,363.52 | 54.50 |
|  |  |  |  |  | ✓ | ✓ | ✓ |  | 4,370.56 | 61.54 |
|  |  |  |  |  | ✓ |  | ✓ |  | 4,371.11 | 62.09 |
|  |  |  | ✓ | ✓ | ✓ | ✓ | ✓ |  | 4,371.82 | 62.80 |

**Table S9. Posterior model estimates from the best-fit model testing the effects of predictors on *L. delicata* detections in Question 4.** CI= credible intervals, ESS = effective sample size.

| Species | Level | Estimate | SE | Lower | Upper | Rhat | Bulk ESS | Tail ESS |
| --- | --- | --- | --- | --- | --- | --- | --- | --- |
| *Lampropholis delicata* | Intercept | 0.59 | 1.01 | -1.36 | 2.55 | 1.01 | 409.34 | 806.48 |
|  | Heathland | -2.48 | 1.48 | -5.42 | 0.38 | 1.00 | 532.69 | 1848.83 |
|  | Rainforest | -1.37 | 2.31 | -5.84 | 3.23 | 1.00 | 819.38 | 1800.85 |
|  | Sedgeland | -3.48 | 2.10 | -7.69 | 0.55 | 1.00 | 717.62 | 1331.56 |
|  | Shrubland | -2.27 | 1.53 | -5.31 | 0.66 | 1.00 | 587.24 | 1011.19 |
|  | Woodland | 0.63 | 1.49 | -2.34 | 3.59 | 1.01 | 516.44 | 1201.21 |
|  | Year | 1.31 | 2.03 | -2.59 | 5.30 | 1.01 | 380.91 | 815.25 |
|  | Late | -2.26 | 1.04 | -4.30 | -0.27 | 1.01 | 403.24 | 942.21 |
|  | Year^2 | 0.58 | 0.93 | -1.23 | 2.42 | 1.01 | 398.18 | 907.77 |
|  | Spring | 1.14 | 0.25 | 0.65 | 1.63 | 1.00 | 2728.48 | 2749.99 |
|  | Summer | 1.13 | 0.25 | 0.64 | 1.63 | 1.00 | 2553.47 | 2487.38 |
|  | Heathland:Year | -0.73 | 2.89 | -6.46 | 4.81 | 1.01 | 510.97 | 1638.82 |
|  | Rainforest:Year | -3.02 | 4.45 | -11.66 | 5.78 | 1.00 | 755.24 | 1737.45 |
|  | Sedgeland:Year | -3.61 | 4.00 | -11.38 | 4.11 | 1.00 | 676.37 | 1479.13 |
|  | Shrubland:Year | -1.62 | 3.00 | -7.62 | 4.04 | 1.00 | 557.52 | 1007.64 |
|  | Woodland:Year | 1.28 | 2.88 | -4.51 | 6.99 | 1.01 | 485.67 | 982.33 |
|  | Heathland:Late | 1.85 | 1.48 | -0.99 | 4.71 | 1.00 | 536.92 | 1605.22 |
|  | Rainforest:Late | 0.11 | 2.34 | -4.63 | 4.57 | 1.00 | 805.56 | 1919.92 |
|  | Sedgeland:Late | 3.90 | 2.11 | -0.15 | 8.15 | 1.00 | 721.45 | 1072.59 |
|  | Shrubland:Late | 1.05 | 1.54 | -1.92 | 4.08 | 1.00 | 600.84 | 1035.78 |
|  | Woodland:Late | -1.36 | 1.49 | -4.31 | 1.59 | 1.01 | 511.24 | 1108.90 |
|  | Year:Late | -2.15 | 2.11 | -6.22 | 1.90 | 1.01 | 414.27 | 931.64 |
|  | Heathland:Year^2 | 0.12 | 1.32 | -2.57 | 2.62 | 1.00 | 531.82 | 1436.83 |
|  | Rainforest:Year^2 | -1.68 | 1.99 | -5.55 | 2.20 | 1.00 | 762.30 | 1744.06 |
|  | Sedgeland:Year^2 | -1.50 | 1.80 | -4.97 | 1.96 | 1.00 | 706.29 | 1487.01 |
|  | Shrubland:Year^2 | -0.79 | 1.36 | -3.46 | 1.82 | 1.00 | 577.81 | 1161.55 |
|  | Woodland:Year^2 | 0.56 | 1.30 | -2.09 | 3.13 | 1.01 | 507.38 | 975.01 |
|  | Late:Year^2 | -0.67 | 0.99 | -2.60 | 1.26 | 1.01 | 434.76 | 874.10 |
|  | Heathland:Year:Late | 1.89 | 3.00 | -3.96 | 7.89 | 1.00 | 555.99 | 1756.78 |
|  | Rainforest:Year:Late | 4.46 | 4.64 | -4.62 | 13.42 | 1.00 | 841.27 | 1818.32 |
|  | Sedgeland:Year:Late | 2.44 | 4.16 | -5.63 | 10.74 | 1.00 | 714.17 | 1457.30 |
|  | Shrubland:Year:Late | 2.70 | 3.13 | -3.39 | 8.91 | 1.00 | 590.54 | 1218.28 |
|  | Woodland:Year:Late | 0.92 | 3.00 | -5.01 | 6.85 | 1.01 | 516.83 | 1041.62 |
|  | Heathland:Late:Year^2 | -0.26 | 1.39 | -2.90 | 2.50 | 1.00 | 562.58 | 1527.93 |
|  | Rainforest:Late:Year^2 | 0.74 | 2.22 | -3.58 | 5.02 | 1.00 | 882.94 | 1934.69 |
|  | Sedgeland:Late:Year^2 | 1.83 | 2.00 | -2.01 | 5.76 | 1.00 | 865.59 | 1757.14 |
|  | Shrubland:Late:Year^2 | 0.66 | 1.47 | -2.12 | 3.57 | 1.00 | 680.82 | 1678.18 |
|  | Woodland:Late:Year^2 | -1.79 | 1.41 | -4.62 | 1.02 | 1.01 | 596.05 | 1289.45 |


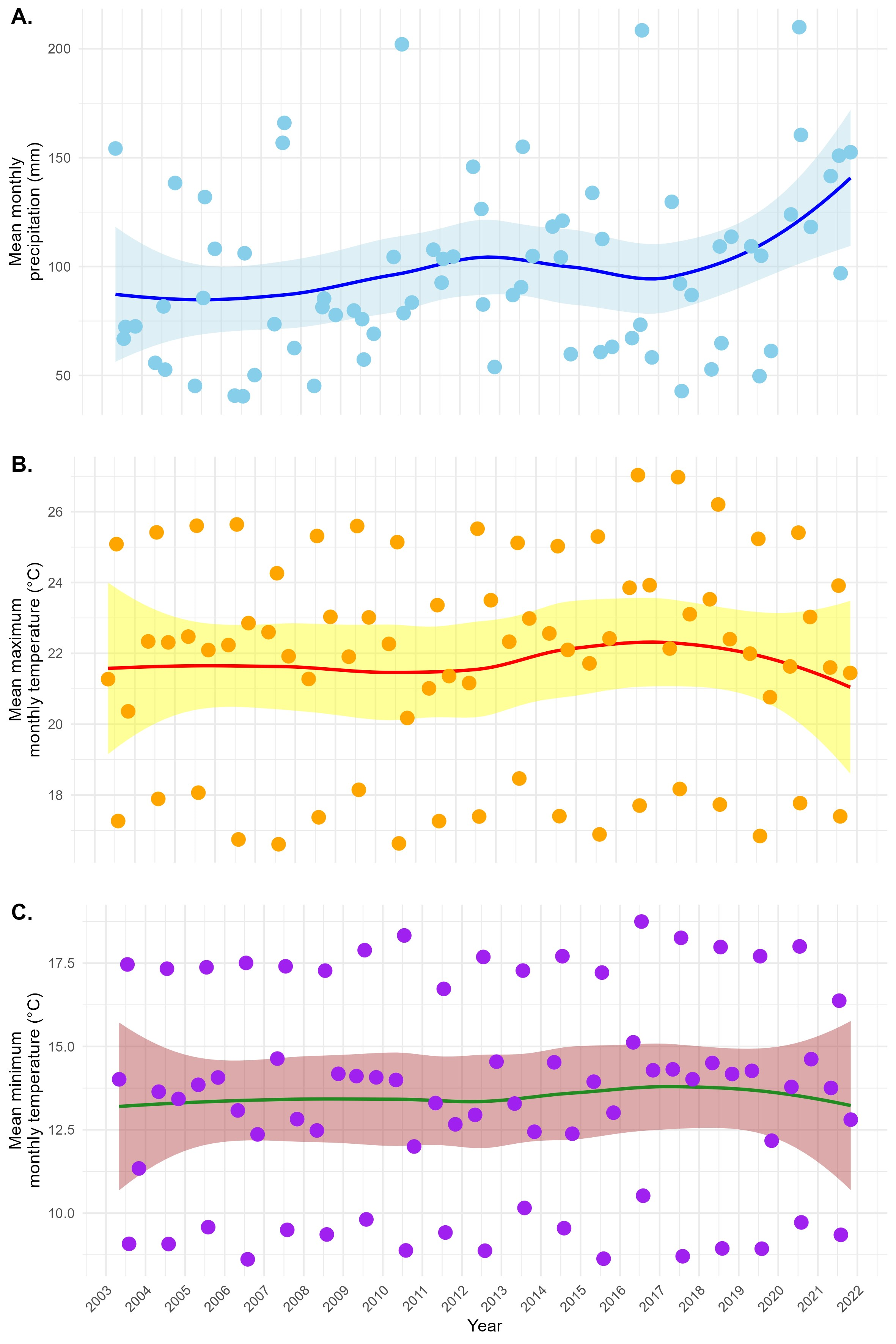


**Fig. S1. Climate trends for BNP during the time of the study.** A. mean monthly precipitation, B. Mean maximum monthly temperature, and C. mean minimum monthly temperature. Lines of best fit with standard errors are calculated from seasonal means for each year, which are plotted as points on each plot.

B.

A.

**
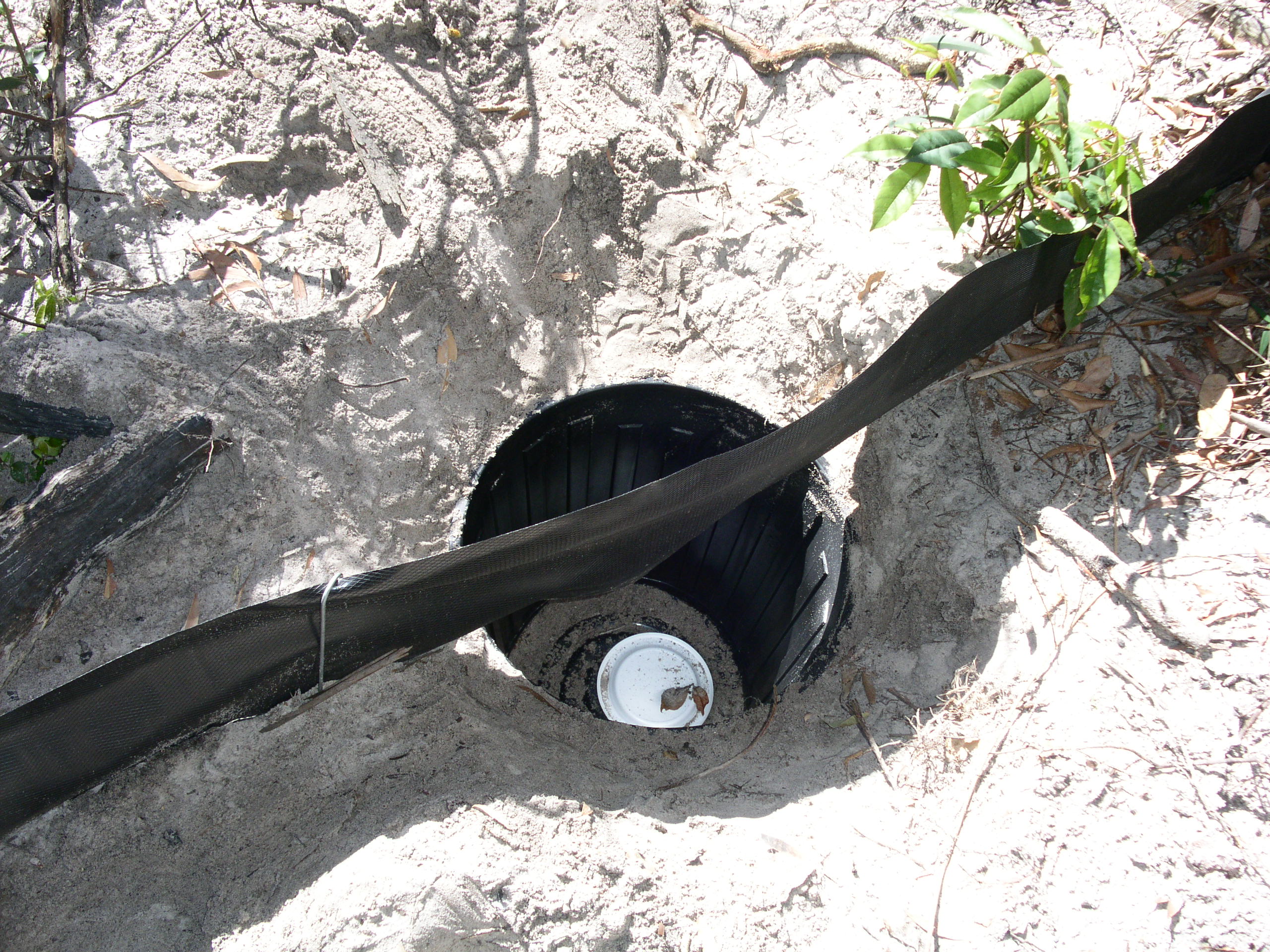
**

**
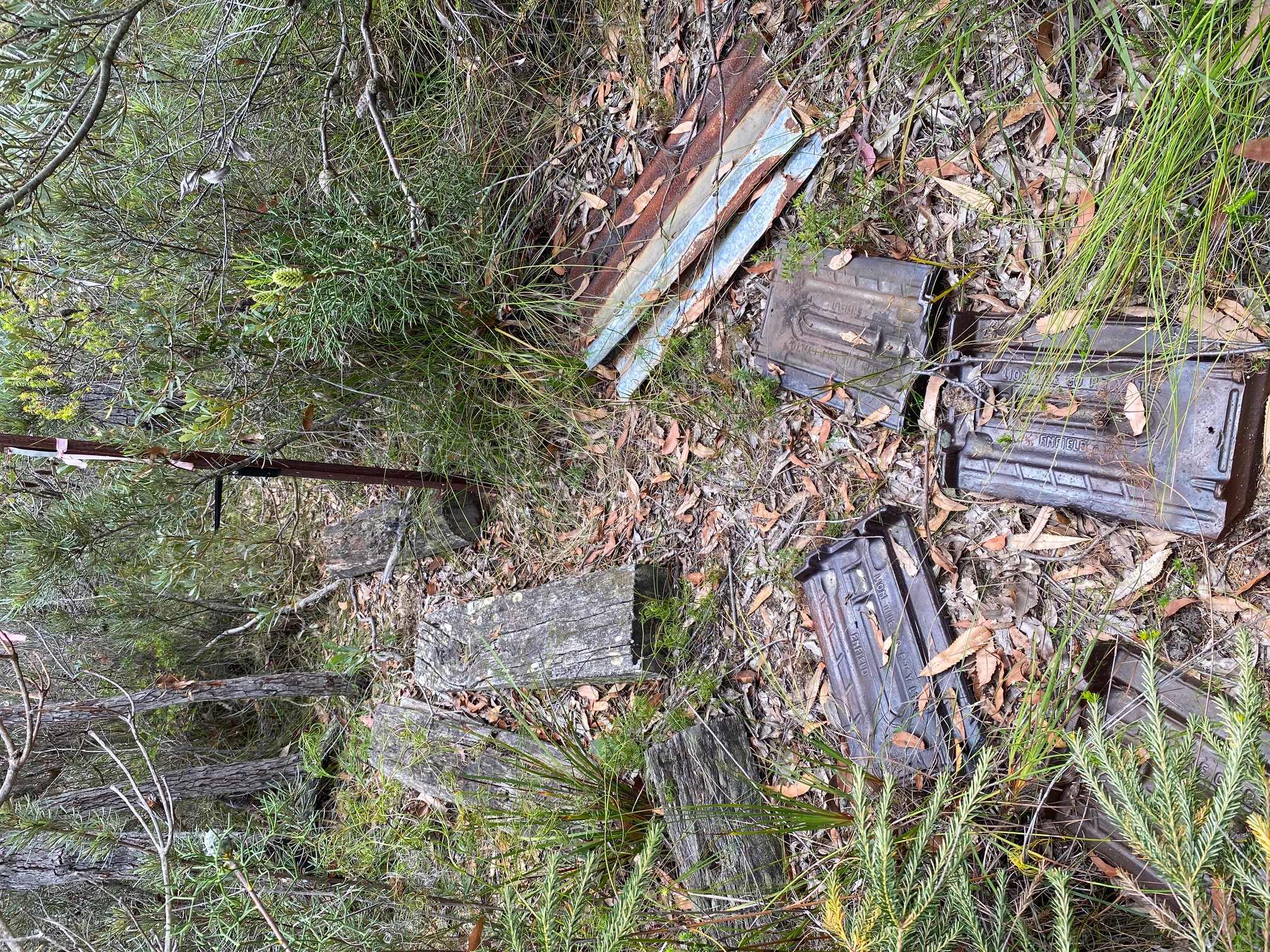
**

**Fig. S2. Examples of (A.) a bucket used in surveys from 2003 until 2010, and (B.) substrates used in surveys from 2011-2022.**


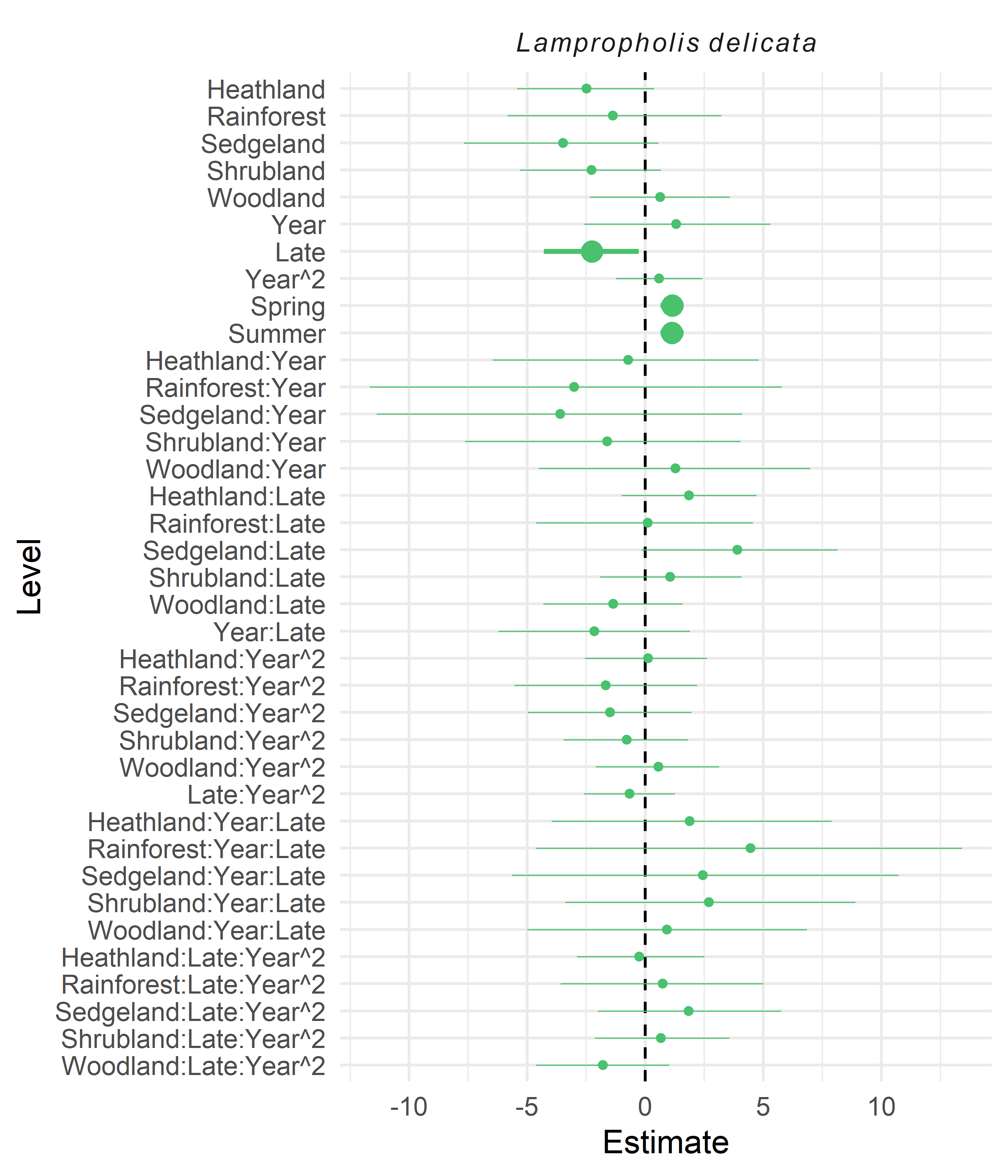


**Fig S3. Predicted plot of *L. delicata* detections in response to time (**$\boldsymbol{Y}_{\boldsymbol{t}}\boldsymbol{)}$**, vegetation type** $\boldsymbol{(V}_{\boldsymbol{i}}\boldsymbol{)}$**, survey methodology (**$\boldsymbol{M}_{\boldsymbol{t}}$**), and their interactions.** Vegetation types (Heathland, Rainforest, Sedgeland, Shrubland, and Woodland) are compared to Forest. Spring and Summer are compared to Autumn. Late is the effect of the late period (2011-2022) to the early period (2003-2010). Error bars represent 95% credible intervals. We considered effects ‘significant’ if their 95% credible intervals did not cross the zero-effect line (larger points). (see Table S10 for model selection results).


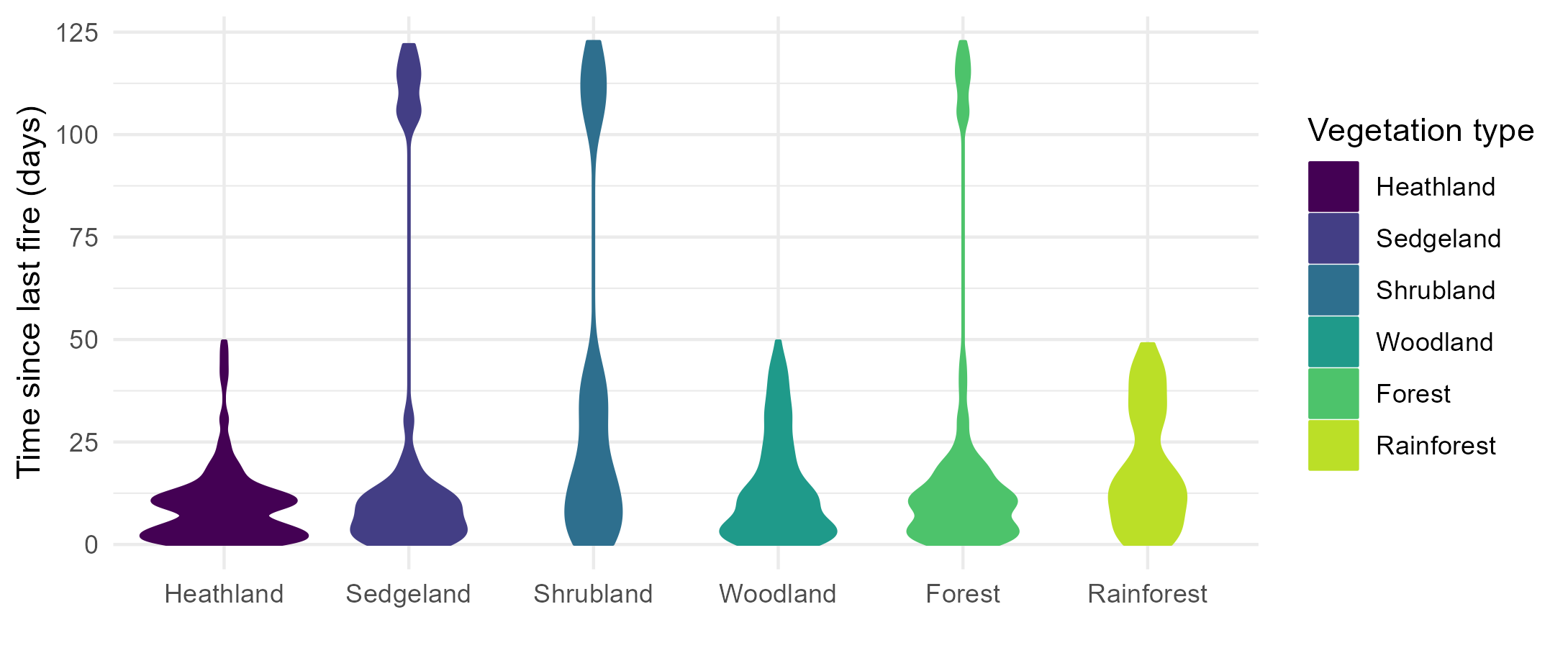


**Fig S4. Violin plots that show the distribution of values for time since the last fire in each of the vegetation types.**
